# Mapping early childhood development data systems in Kenya and sub-Saharan Africa: a scoping review

**DOI:** 10.3389/fpubh.2026.1936644

**Published:** 2026-10-22

**Authors:** Kenneth Okello, George Evans Owino, Evitar Aluoch Ochieng’, Daniel Mwanga, Eubert Munywere, Nelson Langat, Patricia Kitsao-Wekulo, Linda Oloo, Paul Otwate, Calistus Wilunda, Amanuel Abajobir, Silas Onyango, Symon M. Kariuki, Charity Waweru-Mwangi, Margaret Nampijja

**Affiliations:** African Population and Health Research Center, Nairobi, Kenya

**Keywords:** Early Childhood Development data systems, ECD data platforms, nurturing care framework, ECD data challenges, ECD data challenges opportunities

## Abstract

**Background:**

Holistic child development requires a nurturing environment, yet aggregated data on how children fare across the five Nurturing Care Framework (NCF) domains; health, nutrition, early learning, responsive caregiving, and safety and security, remain scarce, especially where vulnerabilities cluster. In sub-Saharan Africa (SSA), the absence of comprehensive, real-time, actionable data impedes timely, evidence-based action to promote healthy development and prevent developmental delays. This scoping review mapped early childhood development (ECD) and ECD-related data systems in SSA, focusing on Kenya and profiling three counties (Nairobi City, Isiolo and Homa Bay).

**Methods:**

Following Arksey and O′Malley’s framework with Levac et al.’s refinements, we searched PubMed, ScienceDirect, African Journals Online (AJOL), Scopus, APA PsycINFO and PsycNet (August 2010–August 2025), plus grey literature from Google Scholar, OpenGrey and national and subnational reports and websites. Of 2,773 records, 2,703 were screened after deduplication and 222 full texts assessed, yielding 49 records describing 44 ECD and ECD-related data systems, sources and platforms. Two reviewers extracted system type, indicators and sources, linkages, stakeholders, and reported challenges and lessons.

**Results:**

Five ECD dashboards were identified: UNICEF’s ECD Dashboard; the National Evaluation Platform (Malawi, Mali, Mozambique, Tanzania); the Tanzanian ECD Dashboard; and two South African platforms, Ecdinfohub.org and HearScreen. Overall, 44 systems were identified across nine countries, as well as regional and LMIC-wide sources; More than half (23/44; 52.3%) were based in Kenya, including five county-specific platforms. Coverage of the Nurturing Care Framework (NCF) domains was uneven: Health indicators were the most commonly represented (30 systems, 68.2%), followed by nutrition (18, 40.9%). In contrast, early learning (13, 29.5%), safety and security (7, 15.9%), and responsive caregiving (6, 13.6%) were substantially less represented. Only eight systems (18.2%) included cross-cutting financing indicators. Most Kenyan platforms were not specifically designed for ECD, and several suffered from irregular updates or limited public accessibility. Common challenges across systems included technical limitations, poor data quality, insufficient capacity, fragmented data ecosystems, and weak links between data and policy processes. Notably, only three platforms and one multi-country survey instrument, the Multiple Indicator Cluster Survey (MICS) covered all five domains, none of them subnationally at child level.

**Conclusion:**

ECD data in Kenya and SSA remain fragmented along sectoral lines, and no single system generates the information on indicators across the five components of NCF. Closing this gap requires an interoperable, standards-based platform that federates rather than replaces sectoral systems, built on an agreed minimum ECD indicator package and explicit metadata governance.

**Systematic review registration:**

https://osf.io/ht24x/overview.

## Introduction

Approximately 250 million children worldwide are at risk of failing to reach their full developmental potential due to persistent poverty, disease, malnutrition, and a lack of nurturing and stimulating environments. (1). The risk of failing to thrive is disproportionately higher in low- and middle-income countries (LMICs), especially in sub-Saharan African nations, where these adversities are concentrated, compared to settings in high-income countries (2). For example, according to World Bank data, in 2020, 56% of Kenya’s urban population lived in informal settlements, which are characterized by a lack of land or tenancy rights, poor amenities, limited access to health and education services, and high rates of unemployment and crime (3). Collectively, these adverse living conditions undermine the growth and development of the world’s most disadvantaged children. Similarly, remote and hard-to-reach rural communities often face unique challenges, including poverty, poor health, and malnutrition, stemming from prolonged droughts and adversely affecting child growth and development. These communities continue to suffer from unequal access to care services, including early childhood development (ECD) interventions. In 2016, 38.3% of Kenyan children aged 3 and 4 did not reach their cognitive and socio-emotional milestones (4). A recent national ECD survey in Kenya indicated that about 25% of children were developmentally delayed (5). In Isiolo and other remote, climate-vulnerable frontier counties, food insecurity remains widespread, with an estimated 28% of children experiencing malnutrition despite ongoing agricultural interventions (5).

The COVID-19 pandemic increased teenage pregnancies, particularly in underserved areas like Homa Bay County, which has a 24% prevalence rate (6), making it a high-risk zone for substandard childcare and development. Early childhood adverse exposures have long-term effects on an individual’s success later in life if not addressed early. This creates significant negative consequences for community growth and prospects. While child survival is vital, it is equally important for children to thrive and reach their full developmental potential, as this ultimately determines their future success and wellbeing. Therefore, creating evidence-based nurturing environments that promote health, growth, development, and holistic wellbeing is essential.

These patterns are regional rather than uniquely Kenyan. The Nurturing Care Framework (NCF), launched jointly by the World Health Organization, UNICEF and the World Bank Group in 2018, positions health, nutrition, responsive caregiving, opportunities for early learning, and safety and security as five interdependent components that must be monitored together rather than sector by sector (7). The measurement strategies available to governments across sub-Saharan Africa (SSA) have not been effectively implemented. Work by the Countdown to 2030 collaboration has shown that reproductive, maternal, newborn and child health tracking in the region continues to rest largely on periodic household surveys, which offer limited subnational comparison with reporting gaps (8), and the Lancet Series on Early Childhood Development has argued that population-level developmental monitoring remains the weakest link in the ECD evidence chain (9). A recent scoping review of nurturing care measurement organized around the five NCF components and covering more than 50 LMICs, found measurement effort heavily concentrated on health and nutrition, with markedly less attention to early learning, responsive caregiving, and safety and security (10). At the same time, the WHO Global Strategy on Digital Health 2020–2025 commits member states to interoperable, person-centred digital health architectures (11), while comparative benchmarking through the Global Digital Health Monitor shows disparity on digital health across the African region, with standards and interoperability among the domains varying most widely between countries (12).

Common system barriers to data on ECD services and care include inadequate financial resources, poor allocation of limited funds, and fragmented reporting mechanisms resulting from a lack of evidence on indicators across the NCF components (3). This is complicated by the ambiguity surrounding the organizational structure responsible for overseeing ECD services, as well as by a lack of coordination and collaboration among the different sectors. Furthermore, there is no dedicated budget allocation for ECD research and care, with funding being indicator- or sector-specific and not accounting for ECD needs in their entirety or relative needs across two or more aspects of ECD. The primary barrier to the system issue is the absence of a coordinated ECD data visualization system or platform that provides aggregated data for performance monitoring and decision-making, including policies, budget allocation, and service delivery, particularly for the most vulnerable children (3).

In SSA, there is a significant lack of aggregated information on how children thrive within the NCF components, especially in communities facing multiple vulnerabilities or adversities. Across the continent, the lack of comprehensive, real-time, and actionable data hampers decision-makers’ ability to implement timely, evidence-based interventions that could prevent developmental delays in the short and long term. Against this background, we conducted this scoping review to profile the existing ECD data systems in SSA, with a deeper dive into Kenya and a special focus on three counties (Nairobi City, Isiolo, and Homa Bay), as part of a project that aimed to develop an ECD data visualization system and platform.

Prior reviews have addressed complementary questions on the measurement tools and indicators used to assess nurturing care across more than 50 LMICs, organizing their synthesis around the five NCF components; their unit of analysis, however, was the instrument deployed in research studies rather than the systems through which governments routinely collect, store and report data on children (10). Other reviews have examined routine health information systems in isolation (13, 14), or have assessed national digital health strengths and weaknesses without a specific focus on children under five. To our knowledge, no previous review has mapped the operational ECD data systems and platforms in use across SSA. That is the question on which the feasibility of the framework as a routine monitoring tool across the five components of NCF ultimately turns, and it is the gap the present review addresses.

Objectives. The primary objective of this review was to map and characterize the ECD and ECD-related data systems, sources and platforms currently in use at regional, national and county levels across SSA, with a deeper dive into Kenya and three contrasting counties (Nairobi City, Isiolo and Homa Bay). Three secondary objectives followed from it: (i) to determine which indicators each system captures within the five NCF domains, together with the tools, software, infrastructure and update frequency involved; (ii) to identify the stakeholders responsible for the use, management, updating and maintenance of each system; and (iii) to synthesize the documented challenges and lessons bearing on the design of an integrated ECD data platform. These objectives were operationalized as 10 sub-questions using the 3WH framework, presented in Table 1.

**Table 1:** Research questions developed based on the 3WH (What, Who, When, How) framework.

What (topic)	Who (population)	When (temporal)	How (methodological)	Location/county	Research question
Sources of ECD-related data	Under five	August 2010–August 2025	Academic and grey literature	Regionally, Kenya and county-specific	What are the key sources of ECD-related data in the region, specifically in Kenya and Nairobi, Isiolo, and Homa Bay counties?
Types of data systems that currently exist and are being used	Under five	August 2010–August 2025	Academic and grey literature	Regionally, Kenya and county-specific	What data systems exist and are used in the three target counties (Nairobi, Isiolo, and Homa Bay) for capturing/recording and reporting ECD and related data? What electronic data systems/ platforms currently capture and report ECD indicator data in your sector (health and nutrition, education, child protection, etc.)? What level of accessibility does the ECD data system provide? Which platforms are used to manage ECD data? Do ECD data systems integrate with other sectoral databases (e.g., health, education, child protection)?
ECD indicators	Under five	August 2010–August 2025	Academic and grey literature	Regionally, Kenya and county-specific	Which specific ECD indicators are collected in the ECD system (the five areas of NCF – Health, Nutrition, Early Learning, Responsive caregiving, Safety and Security)? How frequently is ECD data updated in your system?
Challenges specific to ECD data collection and reporting	Under five	August 2010–August 2025	Academic and grey literature	Regionally, Kenya and county-specific	What challenges are being faced in ECD data management? What are the primary barriers to data accuracy and reliability?

## Methods

This scoping review follows the guidelines developed by Levac et al. (15) and builds on the scoping review framework proposed by Arksey and O’Malley (16). The review protocol was registered on the Open Science Framework (OSF) at https://osf.io/ht24x/overview. Reporting followed the Preferred Reporting Items for Systematic Reviews and Meta-Analyses extension for Scoping Reviews (PRISMA-ScR) checklist, (Supplementary file A). Consistent with established scoping review methodology, no formal critical appraisal or risk-of-bias assessment of the included sources was conducted. The PRISMA-ScR statement (17) treats critical appraisal as optional in scoping reviews, because their purpose is to map the extent, range and nature of the available evidence rather than to synthesize effect estimates or grade certainty. That rationale applies here, because a substantial share of the included sources are grey literature, government reports and platform documentation for which validated appraisal instruments do not exist. The consequence is that no claim is made in this review about the comparative accuracy or quality of the systems described; the absence of appraisal is revisited in the limitations. The five methodological stages followed were: (1) identifying the research question; (2) selecting relevant studies; (3) charting the data; (4) collating and summarizing the results; and (5) reporting the findings.

### Identification of the research question

The scoping review aimed to address the following two main research questions: What are the existing ECD data systems at the local, national, and regional levels, and which stakeholders are involved in ECD data management at each level of government? Given the specificity of the study setting, we used the 3WH framework by Barroso (18), which considers what (topic), who (population), when (temporal), and how (methodology), to refine our research questions, as shown in Table 1. This framework was preferred over the PICO (Population–Intervention–Comparator–Outcome) framework because PICO is designed for clinical intervention research, whereas our objective was to map and profile data systems, an exploratory task better suited to the descriptive 3WH structure.

### Identifying relevant studies

The study team searched for peer-reviewed literature in PubMed, ScienceDirect, OpenGrey, African Journals Online (AJOL), Scopus, APA PsycINFO, and PsycNet from August 2010 to August 2025. The choice of 2010 was guided by the promulgation of the Kenyan Constitution of 2010, which established county governments. The grey literature search utilized We acknowledge that this temporal boundary may have excluded pre-2010 ECD data systems in other SSA countries where no equivalent structural transition occurred in that year; this is noted as a limitation in the Limitations section. Online resources, including Google Scholar as a search engine, as well as national and sub-national websites and reports (such as those from the Ministry of Health, social protection, the Ministry of Education, county governments). The search included qualitative and quantitative evidence on ECD data systems currently used by various government agencies, as well as the access and challenges encountered when using these systems and platforms.

The literature search strategies for each region are described in Table 2.

**Table 2:** Summary of search strategies per region.

Region	Search strategies
SSA	[(“Child” OR “infant” OR “children” OR “ECD”) AND (“health*” OR “education” OR “safety” OR “security” OR “protection” OR “stimulation” OR “care*” OR “nutrition” OR “early learning” OR “sanitation” OR “ECD infrastructure” OR “ECD financing” OR “ECD budget allocation” OR “ECD budget expenditure”) AND (“data” OR “system” OR “platform” OR “dashboard”) AND (“SSA” OR “sub-Saharan Africa”)]
Kenya	[(“Child” OR “infant” OR “children” OR “ECD”) AND (“health*” OR “education” OR “safety” OR “security” OR “protection” OR “stimulation” OR “care*” OR “nutrition” OR “early learning” OR “sanitation” OR “ECD infrastructure” OR “ECD financing” OR “ECD budget allocation” OR “ECD budget expenditure”) AND (“data” OR “system” OR “platform”) AND (“Kenya”)]
Nairobi	[(“Child” OR “infant” OR “children”) AND (“health*” OR “education” OR “safety” OR “security” OR “stimulation” OR “care*” OR “nutrition” OR “early learning” OR “sanitation” OR “ECD infrastructure” OR “ECD financing” OR “ECD budget allocation” OR “ECD budget expenditure”) AND (“data” OR “system” OR “platform”) AND (“Nairobi”)]
Isiolo	[(“Child” OR “infant” OR “children” OR “ECD”) AND (“health*” OR “education” OR “safety” OR “security” OR “protection” OR “stimulation” OR “care*” OR “nutrition” OR “early learning” OR “sanitation” OR “ECD infrastructure” OR “ECD financing” OR “ECD budget allocation” OR “ECD budget expenditure”) AND (“data” OR “system” OR “platform”) AND (“Isiolo”)]
Homa Bay	[(“Child” OR “infant” OR “children” OR “ECD”) AND (“health*” OR “education” OR “safety” OR “security” OR “protection” OR “stimulation” OR “care*” OR “nutrition” OR “early learning” OR “sanitation” OR “ECD infrastructure” OR “ECD financing” OR “ECD budget allocation” OR “ECD budget expenditure”) AND (“data” OR “system” OR “platform”) AND (“Homa Bay” OR “Homabay”)]

All searches were executed between 1st and 20th December 2025 by two reviewers working independently and were re-run on 5th January 2026 to capture any eligible records falling within the search window that had not been indexed after the original execution. Table 2 presents the core concept blocks common to all databases. The complete, database-specific strategies, including controlled-vocabulary terms (MeSH in PubMed, Emtree in Scopus, and APA Thesaurus terms in PsycINFO and PsycNet), free-text field tags, Boolean operators, truncation and proximity operators, applied limits, and the number of records retrieved per database are reported in full in Supplementary file B, following PRISMA-ScR reporting recommendations for literature search reporting. Strings combined four concept blocks (child or ECD population; NCF domain; data system, platform or dashboard; and geography) with the Boolean operator AND, with synonyms within each block combined with OR and truncation (*) applied to capture morphological variants (for example health*, care*). MeSH headings used in PubMed included “Child Development,” “Child, Preschool,” “Health Information Systems”, “Medical Informatics” and “Africa South of the Sahara” and combined with the corresponding free-text terms. No methodological or study-design filters were applied. Reference lists of all included records were hand-searched for additional eligible sources.

### Study selection

We used the following eligibility criteria to select relevant studies to be included in our scoping review:

### Inclusion criteria

All publications from the scientific electronic databases were eligible for inclusion if they met the following criteria:

- Original observational or experimental studies, including quantitative, qualitative, or mixed methods research;
- Scientific (peer-reviewed) studies or grey literature focusing on children aged 0–5 years;
- Scientific (peer-reviewed) studies or grey literature covering data collection or collation for any of the components of the NCF (health, nutrition, opportunities for early learning, responsive caregiving, and safety and security);
- Published from August 2010 to August 2025;
- Participants from SSA;
- Published in English, Kiswahili, or French.

### Exclusion criteria

Studies or grey literature were excluded based on the following criteria;

- Systematic/scoping reviews and meta-analyses;
- Scientific studies or grey literature focusing on children above 5 years only;
- Published before August 2010;
- Participants from other regions outside SSA;
- Not written in English, Kiswahili, or French.

The eligible studies were initially screened based on their titles and then on their abstracts. Of the 2,703 records remaining after removal of duplicates, 2,481 were excluded at the title-abstract screening stage. In the second stage, we conducted a full-text review of the remaining 222 records to assess eligibility against the established inclusion and exclusion criteria; 173 were excluded, resulting in 49 records. Finally, each publication underwent a thorough review. Records were managed and deduplicated in EndNote 2025, and screening was done manually. Title and abstract screening were performed by one reviewer, with a second reviewer independently screening a random 10% sample to verify consistent application of the eligibility criteria; all records advancing to full text were screened independently in duplicate. Disagreements at either stage were resolved by discussion, with a third reviewer adjudicating where consensus was not reached. Because dual screening at the title and abstract stage was applied to a sample rather than to the full record set, a chance-corrected agreement statistic (Cohen’s kappa) was not computed (19); this is acknowledged in the Limitations. Reasons for exclusion at the full-text stage were recorded and are reported in Figure 1. The process for study selection is illustrated in Figure 1.

**Figure 1:**
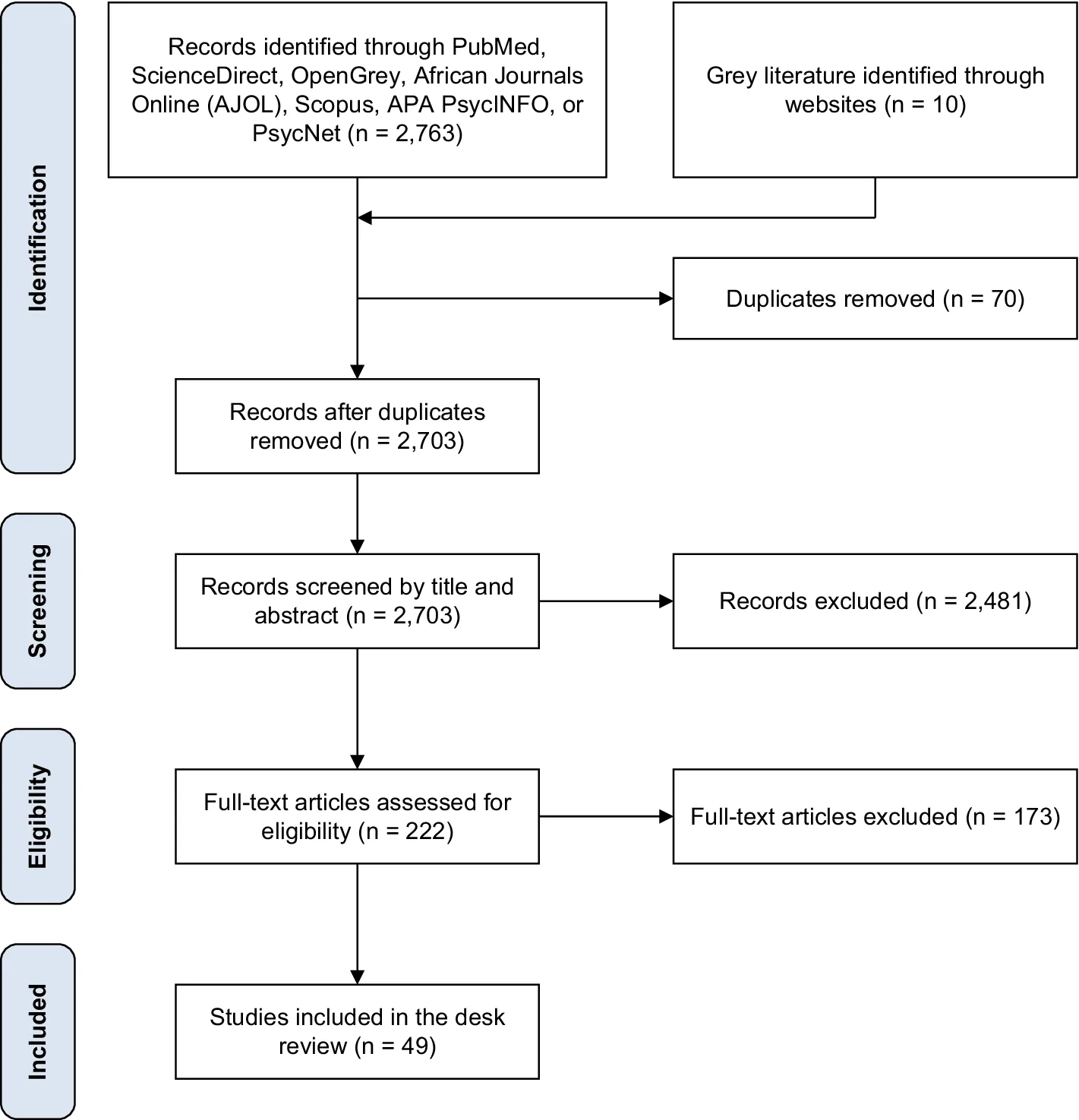
Scoping review flowchart.

### Charting the data

We used the data extraction template in Table 3 to capture information for each study. The data extraction form included the authors’ names, publication date, title, study aims and objectives, settings, population, sampling method, analysis, findings, and conclusion. Extraction variables were aligned with the 3WH framework dimensions: study topic and setting (What/Where), population characteristics (Who), temporal scope (When), and data collection methodology, including software used (How). Reviewers extracted data independently. The extraction form was piloted independently by two reviewers on a random sample of five included records and refined before full extraction, to strengthen the operational distinction between a data source (an instrument, survey or register that generates ECD data) and a data platform (a system that stores, aggregates, links or visualizes such data). Extraction was subsequently performed by one reviewer and independently verified by a second against the source document; discrepancies were resolved by discussion, with a third reviewer consulted where agreement was not reached. No contact with study authors or platform custodians was made to obtain missing information; fields not addressed in a source were charted as “not reported” rather than inferred. Duplicates were identified across the database. Studies reported in different sources were combined during data extraction.

**Table 3:** Data extraction form.

Data platform/source	Author/ Authors	Study site	If Kenya, specify the County	Population (Community-based, hospital-based, school, or household-based)	Study setting (urban or rural)	Study design or type	Sector (Nurturing care framework)	Population age group
								
								
								
								
								
								

### Collating, summarizing, and reporting the results

The research team then mapped evidence and any overall findings associated with the context-specific research questions. Further synthesis was based on the following themes: sources of ECD-related data, types of data systems currently in use, indicators being collected, and challenges specific to data collection and reporting. Synthesis was primarily descriptive, with findings mapped to the five NCF components (health, nutrition, early learning, responsive caregiving, and safety and security) and presented as frequencies and proportions where feasible. Narrative synthesis was used to describe challenges and lessons, grouped thematically across SSA, Kenya national, and county levels.

Synthesis was conducted in three steps. First, a descriptive numerical summary quantified the charted systems by geography, governance arrangement, underlying data source, sector and NCF component coverage, and was reported as frequencies and proportions of the 44 identified systems. Second, a deductive framework synthesis was applied, with the five NCF domains and an ECD resources category serving as *a priori* charting categories; a system was coded as covering a domain if its source documentation reported at least one indicator attributable to that domain. The sectoral label applied in Table 4, and the indicator listings appear in Supplementary files D,E. Domain coding was performed by one reviewer, independently checked by a second, and disagreements resolved by discussion; the resulting platform-by-domain matrix is presented as Table 5. Third, an inductive thematic synthesis of the free-text extraction fields on challenges and lessons was conducted using a constant comparison approach: extracted text was coded line by line, codes were grouped into descriptive categories, and categories were consolidated into the six analytical themes reported below (infrastructure and technical constraints; data quality and completeness; human resource and capacity gaps; systemic fragmentation and limited interoperability; policy and data-use disconnects; and methodological and reporting weaknesses). Coding was managed manually using Microsoft Excel.

**Table 4:** Study characteristics- summary of data systems reviewed.

Data platform/source	Author/authors	Study site	If Kenya, specify the county	Population (community-based, hospital-based, school, or household-based)	Study setting (urban or rural)	Study design or type	Sector (nurturing care framework)	Population age group
Birth history methods	(20)	Zambia		Household survey	Nationwide	Survey	Health	Under 5 years
The Mobile Technology for Community Health (MOTECH)—	(21)	Ghana		Hospital-based	Both rural and urban	Cost-effectiveness analysis	Health	Pregnant mothers and children below 5 years
The Demographic and Health Survey (DHS)	(22–27)	SSA		Household survey	Nationwide	Survey	Health and Education	General population
Tanzania District Health Information System (DHIS2)	(28, 29)	Tanzania		Hospital-based	Both rural and urban	Routine	Health	General population
E-Registry	(30)	Tanzania		Hospital-based	Both rural and urban	Survey	Health	General population
Multiple Indicator Cluster Survey (MICS)	(31–34)	SSA		Household survey	Nationwide	Survey	Health and Education	General population
The World Health Organization’s Global Health Observatory (WHO-GHO) database	(35)	SSA		Household survey	Nationwide	Survey	Health and Education	General population
The NeoTree	(36)	Malawi		Hospital-based	Both rural and urban	Survey	Health	General population
TIER. Net (Three Integrated Electronic Registers)	(36, 37)	South Africa		Hospital-based	Both rural and urban	Survey	Health	General population
The Zambia Electronic Perinatal Record System (ZEPRS)	(38)	Zambia		Hospital-based	Both rural and urban	Survey	Health	General population
Health information system	(39, 40)	SSA		Hospital-based	Nationwide	Survey	Health	General population
The UNICEF dashboard/ countdown 2030	(34, 41)	SSA		Household survey	Nationwide	Survey	Health, Nutrition, Early Learning, Responsive Caregiving, Safety, and Security	General population
Tanzanian Early Childhood Development Dashboard	(42, 43)	Tanzania		Household survey, Hospital-based	Nationwide	Survey	Health, Nutrition, Early Learning, Responsive Caregiving, Safety, and Security	Children 0–5 years
HearScreenTM application [Note: HearScreen™ is a point-of-care hearing screening tool rather than a population-level ECD dashboard. It is classified here under data sources (facility/community-based) rather than platforms.]	(44)	South Africa		School and community-based	Both rural and urban	Survey	Health	Preschool children
The National Evaluation Platform (NEP)	(27)	Malawi, Mali, Mozambique, and Tanzania		Household survey	Nationwide	Survey	Health and Education	General population
The UNESCO Institute for Statistics (UIS) The UIS is the primary source of data for Sustainable Development Goal 4.1, focusing on learning outcomes for children at primary and lower secondary levels. It covers 4- to 5-year-olds in preschool settings and offers internationally comparable, SDG-aligned education indicators. A key limitation is its focus on school-readiness and school-age outcomes rather than children 0–3; any new ECD platform should align with UIS standards while extending indicator coverage to the full 0–5 age range.	(45)	SSA		School and community-based	Both rural and urban	Survey	Education	Preschool children
The Saving Little Lives (SLL)	(45, 46)	Ethiopia		Hospital-based	Both rural and urban	Survey	Health	Mothers and newborns
Education Management Information System (EMIS)	(47, 48)	LMICs		School-based	Both rural and urban	Survey	Education	School-age children
Ghana’s Education Management Information System	(49)	Ghana		School-based	Both rural and urban	Survey	Education	School-age children
Ecdinfohub.org	(50)	SSA		Household survey	Nationwide	Survey	Health, Nutrition, Early Learning, Responsive Caregiving, Safety, and Security	General population
Suite of Early Learning Measurement Tools (ELOM)	(51)	South Africa		School-based	Both rural and urban	Survey	Education	4 & 5 years
Kenya Health Information System (DHIS-2)	(52–54)	Kenya	Nationwide	Hospital-based	Both rural and urban	Survey	Health	General population
Kenya Electronic Medical Records System (EMR)	(53, 55, 56)	Kenya	Nationwide	Hospital-based	Both rural and urban	Survey	Health	General population
Antenatal, Intrapartum and Postnatal Care COVID-19 Surveillance Study (ANCOV)	(53)	Kenya	Nationwide	Hospital-based	Both rural and urban	Survey	Health	General population
Electronic Health Record System (Afya EHMS)	(56, 57)	Kenya	Nationwide	Hospital-based	Both rural and urban	Survey	Health	General population
Demographics Kenya Dashboard	(47)	Kenya	Nationwide	Hospital-based	Both rural and urban	Survey	Health	General population
The Kenya County Health Fact Sheets dashboard	(58)	Kenya	Nationwide	Hospital-based	Both rural and urban	Survey	Health	General population
Integrated Community Case Management (iCCM)	(58, 59)	Kenya	Nationwide	Hospital-based	Both rural and urban	Survey	Health	General population
National Education Management Information System (NEMIS)	(60)	Kenya	Nationwide	School-based	Both rural and urban	Survey	Education	School-age children
Civil Registration and Vital Statistics	(54)	Kenya	Nationwide	Not specified	Both rural and urban	Survey	Safety and security	General population
Child Protection Management Information System	(54)	Kenya	Nationwide	Not specified	Both rural and urban	Survey	Safety and security	General population
Kenya National Bureau of Statistics and the National Treasury of Kenya on resource allocation	(61)	Kenya	Nationwide	Household based	Both rural and urban	Survey	ECD financing	General population
Data visualization portal	(58)	Kenya	Nationwide	Household based	Both rural and urban	Survey	Public finance, health, and education sectors	General population
KenInfo-Kenya Socio-Economic Database	(58)	Kenya	Nationwide	County level	Both rural and urban	Survey	ECD financing	General population
Kenya National Data Archive (KeNADA)	(58)	Kenya	Nationwide	County level	Both rural and urban	Survey	ECD financing	General population
Integrated Management Information System (IMIS)	(58)	Kenya	Nationwide	County level	Both rural and urban	Survey	ECD financing	General population
Wazi Map Kenya Dashboard	(58)	Kenya	Nationwide	County level	Both rural and urban	Survey	ECD financing	General population
Kenya Open Data Platform	(58)	Kenya	Nationwide	National level	Both rural and urban	Survey	ECD financing	General population
The Open County Dashboard	(58)	Kenya	Nationwide	County level	Both rural and urban	Survey	ECD financing	General population
Nairobi Urban Health and Demographic Surveillance System (NUHDSS)	(62)	Kenya	Nairobi	Household	Urban	Survey	Health	General population
Standardized Monitoring and Assessment of Relief and Transition (SMART)	(63)	Kenya	Isiolo	Household	Both rural and urban	Survey	Health	General population
Maternal and Child Health (MCH) booklet	(64)	Kenya	Homa Bay	Hospital-based	Both rural and urban	Survey	Health	General population
Integrated Community Case Management (iCCM)	(59, 65)	Kenya	Homa Bay	Hospital-based	Both rural and urban	Survey	Health	General population
Pediatric Index Testing Cascade in Homa Bay, Kenya	(66)	Kenya	Homa Bay	Hospital-based	Both rural and urban	Survey	Health	General population
Data Quality Audits (DQAs), the Nutrition Information Technical Working Group (NITWG), County Nutrition Technical Forums (CNTFs), and Sub-County Nutrition Technical Forums (SCNTFs)	(67)	Kenya	Homa Bay	Hospital-based	Both rural and urban	Survey	Health	General population

**Table 5:** Comparative summary of the principal ECD and ECD-related platforms, ranked by Nurturing Care Framework domain coverage.

Platform or source	Geographic scope	NCF domains covered	Interoperability with other systems	Public accessibility	Update frequency and operational status
UNICEF ECD Dashboard/Countdown to 2030	SSA (regional)	5 of 5	Aggregates national survey estimates; no routine-system linkage reported	Open, public	Not reported in the reviewed sources; survey-round dependent
Tanzanian ECD Dashboard	National (Tanzania)	5 of 5	Draws on both routine (DHIS2) and survey sources	Open, public	Not reported in the reviewed sources
Ecdinfohub.org	National (South Africa)	5 of 5	Not reported in the reviewed sources	Open, public	Not reported in the reviewed sources
Multiple Indicator Cluster Survey (MICS)	SSA (multi-country)	5 of 5 (survey instrument, not a platform)	Microdata released separately; no routine linkage	Open, public	Periodic survey rounds (typically 3–5 years)
Demographic and Health Survey (DHS)	SSA (multi-country)	4 of 5 (survey instrument, not a platform)	Microdata released separately; no routine linkage	Open, public	Periodic survey rounds (typically 5 years)
National Evaluation Platform (NEP)	Malawi, Mali, Mozambique, Tanzania	3 of 5	Documented difficulty linking to DHIS2 owing to differing analysis software	Restricted	Not reported in the reviewed sources
Kenya Health Information System (KHIS/DHIS2)	National (Kenya)	2 of 5	National HIS gateway; EMR integration documented as incomplete	Restricted; selected aggregate dashboards public	Routine reporting; active
Kenya Electronic Medical Records (EMR)	National (Kenya)	2 of 5	Incomplete direct integration with DHIS2 for routine data	Restricted	Continuous at point of care; active
NEMIS/KEMIS	National (Kenya)	2 of 5	No documented linkage to health or child protection systems	Restricted	Annual school census; partially digitised
NUHDSS	Nairobi City County	2 of 5	Research surveillance platform; not linked to routine systems	Restricted (research access)	Continuous surveillance; active
SMART surveys	Isiolo County	2 of 5	Standalone survey; results reported to nutrition forums	Reports public	Periodic (annual or biannual)
Child Protection MIS (CPIMS)	National (Kenya)	1 of 5	No documented cross-sectoral linkage	Restricted	Case-based; partially digitised
Civil Registration and Vital Statistics	National (Kenya)	1 of 5	Not reported in the reviewed sources	Aggregate reports public	Continuous registration; active
Kenya Open Data Platform/Open County Dashboard	National and county (Kenya)	ECD financing	Not reported in the reviewed sources	Open, public	Not reported in the reviewed sources
KenInfo—Kenya Socio-Economic Database	National (Kenya)	ECD financing	Not reported in the reviewed sources	Open, public	Dormant; last updated November 2013
Wazi Map Kenya Dashboard	National and county (Kenya)	ECD financing	Not reported in the reviewed sources	Open, public	Dormant; based on 2009 census data

## Results

We present a summary of the documents retrieved and selected through the stages leading to the final review. The initial search across all databases yielded 2,773 records, comprising 2,763 identified through electronic databases and 10 through grey literature sources. After removal of 70 duplicates, 2,703 records were screened at the title/abstract stage, of which 2,481 were excluded. The full-text review of the remaining 222 records were assessed for eligibility, and 173 were excluded, resulting in 49 records that met eligibility criteria for this review (see Figure 1 for the PRISMA-ScR flowchart).

Table 4 summarizes the characteristics of the 49 studies included in this scoping review. Most of these studies used routinely collected data, such as demographic and health surveys (DHS) and multiple indicator cluster surveys (MICS). Other studies used facility-based clinical datasets and administrative datasets, such as health information systems (HIS) and education management information systems (EMIS). Exact frequencies and proportions by data source type are presented in Table 4.

The 49 included records described 44 distinct data systems, sources and platforms, several systems being documented by more than one record (Table 4). Geographically, 23 (52.3%) were Kenyan, 18 operating nationwide and 5 county-specific (three in Homa Bay, one in Nairobi City and one in Isiolo), while 21 (47.7%) were located elsewhere in SSA, covering nine individual countries (Ethiopia, Ghana, Kenya, Malawi, Mali, Mozambique, South Africa, Tanzania and Zambia) alongside region-wide and LMIC-wide sources. By population base, 18 (40.9%) drew on facility or hospital records, 12 (27.3%) on household surveys, 6 (13.6%) on school or school-and-community platforms, and 6 (13.6%) on county- or national-level administrative and fiscal returns, with the population base not specified for the remaining 2. In terms of governance, most programs were led by government ministries, primarily the Ministries of Health, Education, and Labour or Social Protection, while a smaller proportion were managed or co-financed by multilateral organizations and non-governmental partners.

Coverage across the NCF components was markedly uneven (Figure 2; Table 5). Health indicators were reported by 30 of the 44 systems (68.2%) and nutrition indicators by 18 (40.9%), reflecting the long-standing dominance of routine health information systems and anthropometric survey modules. Early learning indicators appeared in 13 systems (29.5%), almost all of them education sector platforms reporting enrolment or school readiness rather than developmental status. Safety and security indicators were reported by 7 systems (15.9%) and responsive caregiving by only 6 (13.6%), making the latter the least measured of the five components across the region. A further 8 systems (18.2%) reported indicators in the cross-cutting category recorded as “Other: financing,” chiefly budget allocation and expenditure, with no system capturing physical ECD infrastructure such as facility counts or equipment. This category is reported separately from the five NCF components throughout because financing is a cross-cutting enabler of nurturing care rather than a component of it; resources, including human resources, are implied within each of the five components and are not confined to this category. Three platforms, the UNICEF ECD Dashboard, the Tanzanian ECD Dashboard and Ecdinfohub.org, together with one multi-country survey instrument, the Multiple Indicator Cluster Survey (MICS), reported indicators spanning all five NCF components; the DHS covered four. None of these disaggregated child-level indicators across all five domains at the subnational level, meaning that existing monitoring tools at present cannot be mapped against the NCF for a holistic tracking of ECD through any single system in Kenya or across SSA.

**Figure 2:**
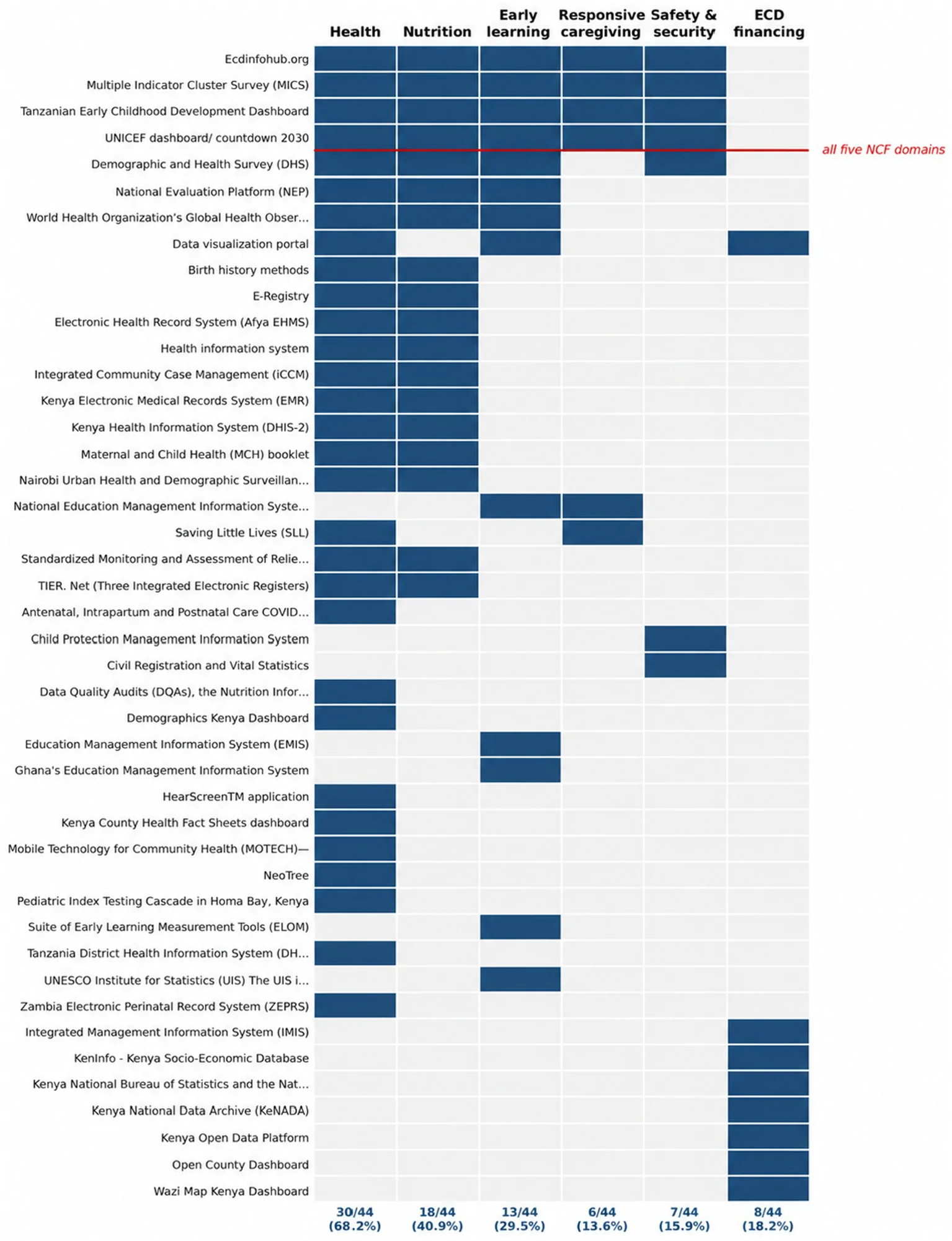
Nurturing Care Framework domain coverage across the 44 charted ECD and ECD-related data systems and sources (shaded = domain covered).

### Sub-Saharan Africa

#### Identified data systems

##### Sources of data

The source of ECD data in SSA related to child health and nutrition ranged from routinely collected data to those gathered at the point of treatment. The routinely collected data included demographic surveys such as the DHS data, followed by the MICS and Health Information Systems, Birth History Methods, Mobile Technology for Community Health (MOTECH), the Demographic and Health Survey (DHS), the Multiple Indicator Cluster Survey (MICS), and the World Health Organization’s Global Health Observatory (WHO-GHO) database. The data collected at the point of treatment include the E-Registry in Tanzania, the NeoTree application in Malawi, TIER. Net (Three Integrated Electronic Registers) in South Africa, the Zambia Electronic Perinatal Record System (ZEPRS), and the Health Information System in most countries in the Southern African region.

Birth history methods: In Zambia, two types of birth histories are routinely captured, which allow for the estimation of under-5 mortality over time in the years preceding a survey or census: complete birth histories (CBH) and summary birth histories (SBH). In a CBH, women are asked detailed questions about each child they have given birth to, including the date of birth, survival status, and (when applicable) age at death. In contrast, in an SBH, women are asked only for the total number of children they have given birth to and the number of these children that have died (20). Birth history data are collected primarily via paper-based household survey questionnaires (DHS/Census enumeration), not through digital capture platforms.

The Mobile Technology for Community Health (MOTECH): MOTECH was a partnership between Grameen Foundation and Ghana Health System (GHS) aimed at enhancing service delivery and access to Maternal, Newborn and Child Health (MNCH) services through two components: Mobile Midwife and Client Data Application (CDA). The Mobile Midwife is a demand-side intervention that aims to enhance client knowledge and awareness of essential health information during pregnancy and the postpartum period, promote best practices, and encourage the timely and appropriate use of health services (21).

The DHS is conducted in over 90 countries and serves as the primary data source for regions with the highest burden; however, concerns regarding data quality persist (23). The DHS is collected periodically every 5 years (24). In East Africa, studies have utilized DHS datasets to analyze the nutritional status of children under 5 years old (68), with findings indicating that most countries have experienced a significant reduction in severe forms of anaemia in recent years (26). Within DHIS 2, data elements are organized by geographic unit, such as district or region. The data may be combined into indicators and displayed through tables, figures, or maps within the software (25). DHIS 2 requires relatively minimal training to use (22). An advantage of open-source software is that external applications (apps) can be developed to introduce new functionality to the core platform (27).

Tanzania District Health Information System (DHIS2): The Tanzania DHIS2 incorporates newborn indicators reported at the facility level. Furthermore, the advancements in DHIS2, utilizing digital technology, along with easier integration and linkage to web-based systems, have presented significant opportunities for data access and availability, enabling data analysis to monitor trends at the facility level against established targets and global standards (28, 29).

Multiple Indicator Cluster Survey (MICS): The MICS is a nationally representative, cross-sectional household survey conducted every 5 years in LMICs (33), covering a range of indicators from health to safety and security (34). The data focus on household assets for safety and security (69). Notably, UNICEF has utilized these household surveys to create an ECD data dashboard, which illustrates progress in each ECD indicator (34). In other studies, MICS has been used to develop a Multidimensional Early Child Development (MECD) framework that encompasses several aspects of a child’s wellbeing, including literacy, learning ability, social, and physical development (31). This index assesses children’s ability to read, count, recognize numbers, interact with peers and others, their health, physical skills, as well as their ability to follow rules and be independent (32).

The World Health Organization’s Global Health Observatory (WHO-GHO) database: The WHO-GHO used in the study by Arhin et al. (2025) is a freely available dataset containing immunization variables that the WHO routinely collects (35). The World Health Organization defines full immunization for one-year-olds as infants who receive three doses of oral polio vaccine (OPV), three doses of each pentavalent (i.e., diphtheria, tetanus, pertussis, *Haemophilus influenzae*, and hepatitis B) vaccines, one dose of measles-containing vaccine (MCV), and one dose of Bacillus Calmette–Guerin (BCG) vaccine before age one (35).

The NeoTree application was developed and implemented in collaboration with key stakeholders and healthcare workers in Malawi. This is a fully integrated Quality Improvement (QI) tool for hospital-based sick and vulnerable newborns, combining immediate digital data capture, evidence-based algorithmic clinical decision support, and newborn education on one platform. This mHealth app is used on low-cost Android tablets or phones at the hospital bedside by healthcare workers (HCWs) to support the care and treatment of sick newborns. The NeoTree operates offline and synchronizes with the network when it is available. Additionally, it is a not-for-profit venture with open-source code available (36). Results showed improvements in clinical skills, critical thinking, confidence, standardization, and quality of care, reflecting the comprehensive and holistic nature of the NeoTree, task-shifting, and newborn survival initiatives.

TIER.Net (Three Integrated Electronic Registers): this encompasses the following datasets: eHealth, Demographic and Health Information System (DHIS), LABTRAK (NHLS), Pre-ART register, TB sputum register (also known as the TB suspect register), Primary Healthcare (PHC) comprehensive tick register, Daily clinic register, HCT register, TB register, Notification of medical condition (TB), NHLS lab specimen shipping book, Non-standard registers (which differ across facilities), Counselling session register, Tracing register, Adherence club register, Isoniazid preventive therapy (IPT) register, Chronic register, Cryptococcal test register, Standard stationery within patient files for HIV patients on treatment, Chronic patient record, TB treatment record, TB screening tool, and Appointment/carrier cards (37).

The Zambia Electronic Perinatal Record System (ZEPRS): The ZEPRS application utilizes real-time data entry, allowing registered users (e.g., nurses, midwives, and clerical staff) to input information directly from each patient visit at the point of care. Pre-programmed checks prevent the entry of nonsensical values; abnormal (but plausible) findings trigger alerts, enabling providers to correct data entries if mistakes are made or intervene as necessary (38).

E-Registry: This registry was envisioned to enable regular feedback to maternity care providers and enhance the use of facility-level data in Tanzania. On this platform, women’s data are collected in electronic patient records (EPRs) on tablets, thereby providing individual data in real-time. Additionally, to facilitate more straightforward data interpretation by hospital teams, run charts (plots displaying data in chronological order) were shared with the team monthly on this platform. Regarding the quality improvement cycles at the hospitals, the run charts focused on target areas for improvement, such as the completeness of obtaining admission data and the consistency of fetal monitoring (30).

Health information system: This includes community reports transmitted from district health centers at the point of treatment or care (40). To strengthen HIS, some countries have introduced digital data collection (DDC) tools to support field data collection and management for a wide range of health interventions. In the case of malaria, mobile DDC tools have been utilized to collect data on malaria case management, vector control, clinical trials, national surveillance, and household surveys. Such DDC tools included CommCare, iFormBuilder, and Red Rose (39). Across these data sources, several shared characteristics emerge: (1) health and nutrition indicators predominate, with early learning, responsive caregiving, and safety/security rarely collected in the same system; (2) community-based systems (DHS, MICS) are periodic rather than continuous; (3) the DHIS2 platform serves as the most common shared digital infrastructure, suggesting it could anchor a future integrated ECD data hub.

Suite of Early Learning Measurement Tools (ELOM)—The design of the ELOM child assessment tools aims to produce affordable and robust instruments aligned with South Africa’s National Curriculum Framework for children from birth to 4 years, the Grade R (kindergarten) Curriculum Assessment Policy Standards (CAPS) (the ELOM 4 & 5 Years Assessment Tool), and the Grade 1 mathematics and language CAPS (the ELOM 6 & 7 Literacy and Mathematics Assessment Tools). A further objective is to develop tools that can be administered by trained non-professionals in under an hour and provide equitable assessments of children across the ethnolinguistic and socio-economic spectrum. The ELOM 4&5 Years Assessment Tool is designed to assess whether children in the age group of 50–69 months are ready to learn in Grade R. The tool measures functioning in five domains: Gross Motor Development (GMD), Fine Motor Coordination and Visual Motor Integration (FMC-VMI), Emergent Numeracy and Mathematics (ENM), Cognitive and Executive Functioning (CEF), and Emergent Literacy and Language (ELL). Most of the 23 items in this tool were derived from existing instruments and are aligned with the developmental outcomes specified in the National Curriculum Framework for children from birth to 4 years (51). The ELOM offers four key transferable design principles for future ECD platforms: (1) alignment with national curriculum frameworks; (2) non-specialist administration in under 1 h; (3) multi-domain neuropsychological coverage (gross motor, fine motor, emergent numeracy, executive function, emergent literacy); (4) predictive validity for Grade R readiness, providing longitudinal utility. The ELOM offers four key design principles transferable to a future ECD data platform: (1) alignment with national curriculum frameworks (anchored to South Africa’s NCF, demonstrating context-grounded instrument design); (2) non-specialist administration in under 1 h, reducing reliance on scarce specialist personnel; (3) multi-domain neuropsychological coverage (gross motor, fine motor, emergent numeracy, executive function, and emergent literacy in a single instrument); (4) demonstrated predictive validity for Grade R readiness, providing longitudinal utility beyond cross-sectional monitoring.

The data sources in this section span three modalities: (1) community/household-based (DHS, MICS, birth history surveys); (2) facility-based (E-Registry, NeoTree, TIER. Net, ZEPRS, DHIS2, EMR); (3) hybrid systems with community–facility linkages (HIS, iCCM).

##### Data platforms

Examples of such data platforms and dashboards on child health and nutrition in SSA include the UNICEF dashboard, the Tanzanian Early Childhood Development Dashboard, the HearScreenTM application in South Africa, and the National Evaluation Platform (NEP) in Malawi, Mali, Mozambique, and Tanzania. On opportunities for early learning, the results showed several data platforms used for early learning and responsive caregiving, such as the UNESCO Institute for Statistics (UIS), Saving Little Lives (SLL) in Ethiopia, the Education Management Information System (EMIS) used in most LMICs, Ecdinfohub.org, and the ELOM in South Africa.

The UNICEF dashboard utilizes the MICS surveys to create an Early Childhood Development (ECD) data dashboard. MICS is a nationally representative, cross-sectional household survey conducted every 5 years in LMICs, which covers a range of indicators from health to safety and security (50). The dashboard presents information on each country’s data, illustrating progress in each ECD indicator (34).

Tanzanian Early Childhood Development Dashboard: the ECD dashboard is an app currently in development; it includes indicators from the five NCF components: Health, Nutrition, Early Learning, Responsive Caregiving, and Safety and Security (42). This platform offers a straightforward and intuitive way to access and visualize key selected (priority) ECD indicators, providing a graphical user interface that enables users to interact with it easily (42). The data are drawn from multiple data systems (sources), including the DHS from various ministries, such as health, education, and others that have ECD data.

HearScreenTM application: the HearScreen™ mHealth solution enables the use of pre-specified screening protocols with automated sequences by non-specialist personnel. This means that generalist healthcare workers or schoolteachers can be trained to operate the device, after which patient-specific data and results collected through the smartphone application can be uploaded to a centralized cloud-based server via cellular networks. This enables asynchronous point-of-care diagnostics in hard-to-reach populations, with cloud-based data management, surveillance, and referrals, which in turn may reduce the demand placed on already limited professional ear and hearing health human resources in developing countries. The HearScreen™ software utilizes noise monitoring algorithms that provide operators with real-time feedback on ambient noise levels, thereby serving as a guide to minimize the impact of noise levels when testing in varying noise conditions (44).

The National Evaluation Platform (NEP): The NEP concept involved developing an integrated set of data organized by geographic area, drawing from a variety of data sources available in the country, including household surveys (Demographic Health Surveys, Multiple Indicator Cluster Surveys, etc.), facility surveys (Service Provision Assessment, etc.), and administrative sources (27). The NEP data include core indicators from the maternal, newborn and child health and nutrition (MNCH&N) program impact pathways. They are updated as new data are released, facilitating rapid analysis in response to time-sensitive questions from MNCH&N stakeholders (27). The NEP platform was funded by the Canadian government and is implemented by public-sector stakeholders in four countries in SSA: Malawi, Mali, Mozambique, and Tanzania.

The UNESCO Institute for Statistics (UIS)—It is the primary source of data for Sustainable Development Goal 4.1, which focuses on ensuring that all children achieve at least a minimum proficiency level in reading and mathematics by the end of primary and lower secondary education. The UIS collects and disseminates data on various aspects of SDG 4, including indicator 4.1.1, which measures the percentage of children and young people achieving minimum proficiency levels in reading and mathematics at different stages of education (45).

The Saving Little Lives (SLL)—This is an initiative implemented in Ethiopia, utilizes DHIS2 data, specifically those related to the uptake of Kangaroo Mother Care (KMC) and the provision of skin-to-skin contact (46).

Education Management Information System (EMIS)—EMIS is a system that collects, analyses, disseminates, and utilizes education-related data. It is implemented across most LMICs (48). The primary objective of such systems is to provide systematic and high-quality data, enabling countries to formulate and implement education policies based on evidence, thereby improving learning outcomes (47).

Ghana’s Education Management Information System—Lists all registered schools, both private and public, that meet the Ministry of Education quality standards (49).

**Ecdinfohub.org**—This is the Data and Statistics section of the ECD Info Hub, specifically focusing on South Africa’s ECD status. This data platform provides comprehensive and up-to-date information on the state of early childhood development throughout South Africa, supported by reliable data and insightful statistics. The platform aims to equip stakeholders with the information necessary to understand the landscape of ECD, which is crucial for informed decision-making and policy formulation to promote the wellbeing and development of the nation’s youngest citizens. It also presents visualizations that illuminate various aspects of ECD, including data on access to services, quality of care, and outcomes for children (50).

##### ECD indicators included in each data platform/system

Depending on their scope, the platforms cover a range of ECD indicators across the NCF. Health systems/platforms tend to have many indicators on children. Notably, there were overlaps among the platforms in the types of indicators they covered. The complete indicator listing for each data source and platform identified in SSA is provided as Supplementary file D.

### Kenya

#### Identified data systems

##### Sources of data

The sources of data related to child health and nutrition in Kenya ranged from routinely collected information to data gathered at the point of treatment. Routinely collected data included demographic surveys such as the Kenya Demographic and Health Survey (KDHS). Data collected at the point of treatment included systems like the Kenya Health Information System (DHIS-2), the Kenya Electronic Medical Records System (EMR), and the Kenya Demographic and Health Survey (KDHS).

Kenya Health Information System (DHIS-2)— In public sector facilities in Kenya, routine service delivery data are captured in paper-based, standardized registers located at various service delivery points within the health facilities. This primary data is then aggregated in paper-based monthly facility reports, which are submitted to the sub-county health records information officer (HRIO) and manually entered into DHIS 2 (52), an open-source web-based repository housed at the national level (53). The KHIS aggregates data collected from registers at various service delivery points (54).

Kenya Electronic Medical Records System (EMR)—The Kenya EMR was customized from the open-source ‘OpenMRS’ platform, which is a software designed for use by LMICs as national electronic medical record systems. It captures patient-level data in real time and aligns with the MOH’s national data collection and reporting tools (56). Data from the Kenya Electronic Medical Record (EMR) is aggregated at the national level through the National Data Warehouse (NDWH) (55), an online repository for individual-level data from EMR sites and other repositories (56). This system is primarily used for HIV care and treatment services among people living with HIV (PLHIV) (53).

Kenya Demographic Health Survey—The sample for the most recent KDHS was drawn from the Kenya Household Master Sample Frame (K-HMSF), which has been developed from the Kenya Population and Housing Census. This is the frame that the KNBS currently uses to conduct household-based sample surveys in Kenya (70). The KDHS is collected every 5–10 years, with the most recent KDHS conducted in 2022.

Civil Registration and Vital Statistics—The source of data on children’s safety and security in Kenya was the Civil Registration and Vital Statistics, which records data on deaths and births as they happen. Registration of births and deaths (54).

##### Data platforms

In Kenya, child health digital platforms include the Antenatal, Intrapartum, and Postnatal Care COVID-19 Surveillance Study (ANCOV), the Electronic Health Record System (Afya EHMS), the Demographics Kenya Dashboard, the Kenya County Health Fact Sheets dashboard, and the Integrated Community Case Management (iCCM) platform. In addition, for the opportunities for early learning the results showed that the only data platform used for early learning and responsive caregiving in Kenya was NEMIS, which KEMIS later replaced.

Antenatal, Intrapartum and Postnatal Care COVID-19 Surveillance Study (ANCOV)—This study is a Bill and Melinda Gates Foundation-funded partnership between the US Centers for Disease Control and Prevention (CDC), Kenya Medical Research Institute (KEMRI), and Kisumu County. ANCOV developed a maternal surveillance platform that leverages the existing research infrastructure established through a long-standing community-based Health and Demographic Surveillance System (HDSS) and the Child Health and Mortality Prevention Surveillance (CHAMPS) study in Kenya to rapidly assess maternal and infant outcomes in the context of COVID-19 (53).

Electronic Health Record System (Afya EHMS): This platform provides a comprehensive picture of patient care from the lowest level of the health system to referral facilities. It was led by the MOH, supported by the WHO, and was referred to as the country’s electronic health record (CEHR) system, now called AfyaEHMS (57). The data in this platform is collected routinely at the point of healthcare service delivery (71).

Demographics Kenya Dashboard—The Demographics Kenya dashboard is an open online platform for visualizing and analyzing demographic variables and socioeconomic characteristics of the population at the county level. The data used in the dashboard are based on the 2009 Kenya Population and Housing Census obtained from the Kenya Open Data platform (58). Data source: Kenya Demographic and Health Surveys.

The Kenya County Health Fact Sheets dashboard—This was created by the Health Policy Project (HPP), which has now been replaced by the Health Policy Plus (HP+) Project, in collaboration with Kenya’s MOH. The dashboard is open-access and provides summary statistics, displayed on maps and graphics, for selected health indicators in all 47 counties (58). It further gives comparisons of the indicators with national estimates. Summary data include: population of males and females, children under 5 years and 1 year; nutrition: percentage of underweight or stunted children; fully immunized children aged 12–23 months; maternal health: births delivered at health facility, contraceptive prevalence; HIV, TB, and Malaria; health personnel (per 100,000 people) in public hospitals; number of health facilities and health care financing. The Fact Sheet derives data from the following primary sources:

- Kenya Demographic and Health Surveys
- Kenya Health Information System (HIS)
- Integrated Disease Surveillance Reports
- Master Facility List

Integrated Community Case Management (iCCM)—Kenya’s primary health care system aims to provide essential health care based on practical, scientifically sound, and socially acceptable methods and technologies, made universally accessible to individuals and families in the community through their full participation, and at a cost that the community and country can afford. The iCCM services are built upon a functional community health system, which in turn leverages and links with the network of primary healthcare facilities (59).

National Education Management Information System (NEMIS)—This is a web-based system used in Kenya to manage educational data and information from schools. Headteachers enter the information on this platform. The system collects data, processes it, and reports on various indicators, ultimately facilitating effective management of the education sector (60). Furthermore, the Ministry of Education has transitioned from NEMIS to the Kenya Education Management Information System (KEMIS) to address the challenges posed by the NEMIS platform.

Child Protection Management Information System—The results showed that only one dashboard provides information on children’s safety and security, namely the Child Protection Management Information System. Tracks data on cases of violence against children, caregiving, adoption, and foster care (54).

ECD financing (budget allocation, budget expenditure and infrastructure): the reviewed literature addressed predominantly budget allocation and expenditure. Physical ECD infrastructure data (facility counts, equipment) were not systematically captured in reviewed platforms, representing a significant monitoring gap.

##### *Sources* of data

The source of data on ECD resource allocation in Kenya is the Kenya National Bureau of Statistics and the National Treasury of Kenya.

Kenya National Bureau of Statistics (KNBS) and the National Treasury of Kenya on resource allocation—Based on these sources of information, non-state actors have assessed the commitment of county governments to ECD expenditure (61). ECD expenditure was evaluated against county budgets from June 2014 to June 2020. Over the last six fiscal years, counties have, on average, allocated between 9.1% of their budgets towards ECD. However, the ECD expenditure provided can best be viewed as a proxy, as the data are combined with several other sectors, such as ICT, Youth Affairs, and Sports & Social Services, making it difficult to ascertain the exact expenditure on ECD alone. UNICEF recommends that governments spend at least 10% of their budgets on education.

##### Data platforms

The KNBS is a semi-autonomous government agency under the Ministry of National Treasury and Economic Planning. The KNBS is the principal government agency in Kenya responsible for collecting, analyzing, and disseminating statistical data; maintaining a comprehensive and reliable national socio-economic database; and establishing standards and methods for the production and dissemination of statistical information across the national statistical system (58). The KNBS has data portals that provide online databases and offline sources. The data sharing portals/platforms are described below:

Data Visualization Portal—This portal contains data on agriculture, public finance, health, and education sectors. Summary data for these sectors is presented in charts and graphs that can be used for quick comparisons and trend analysis.

Kenya National Data Archive (KeNADA)—The KeNADA portal offers datasets from surveys of individual respondents and households (60) collected by the Bureau since 1962, including censuses, integrated budget household surveys, and surveys on HIV and malaria. It provides users with tools to facilitate further analysis and interpretation of the primary data. The primary data from the surveys are available upon request and could support a multi-sectoral analysis to better understand the factors associated with poor nutritional status.

Integrated Management Information System (IMIS)—The IMIS, funded by UNFPA, is an open web-based platform focused on population issues. It is a collection of several statistical databases using data from population censuses and household surveys. The IMIS operates on RETADAM (Retrieval of Data for Small Areas by Microcomputer), an interactive computer system for data processing, analysis, and web dissemination of outputs at national and county levels. The outputs generated are in the form of cross-tabulations, graphs, and maps for use at the county level. This system is currently under development, and capacity-building efforts are being conducted to enable countries to utilize the system (58).

Wazi Map Kenya Dashboard is an online platform that provides easy and quick access to information disaggregated at both national and county levels. It utilizes secondary data from the Kenya National Bureau of Statistics (KNBS) and other sources. The summary data on key variables, including household characteristics, agriculture, education, employment, health, and nutrition (with z-scores for stunting, wasting, and underweight), are displayed in charts and graphs. Wazimap Kenya enables side-by-side comparisons of variables, thereby enhancing the understanding of disparities among administrative areas (58).

Additionally, other data platforms span multiple sectors, including resource allocation, such as the Kenya Open Data Platform and the Open County Dashboard.

Kenya Open Data Platform—This online platform, under the Information and Communication Technology (ICT) Authority of Kenya, provides access to development, demographic, and statistical data for researchers, policymakers, and the general public. The platform will include information on agriculture, health and social data, economy and finance, education, environment, and water and sanitation (58).

The Open County Dashboard—This is an open-access summary information platform developed by the Open Institute in partnership with the Council of Governors (CoG) of Kenya and the World Bank Accountable Devolution Programme, with funding from UKAid and the Australian Department of Foreign Affairs and Trade. The dashboard aims to strengthen county governments’ efforts to be transparent and accountable to their citizens by publishing county-level development data. The dashboard presents summary statistics on agriculture, rural development, health, security, education, energy, science and technology, and budget indicators by county (58).

### Nairobi City County

#### Identified data systems

##### Sources of data

The sources of data on child health and nutrition in Nairobi City County were routinely collected data from the Nairobi Urban Health and Demographic Surveillance System (NUHDSS) by the African Population and Health Research Centre (APHRC). Notably, there were no ECD Data platforms or sources on other components of the nurturing and care framework, such as responsive caregiving, safety, security, or ECD resourcing.

The NUHDSS was established by the APHRC in 2002 within two slum communities in Nairobi city (Korogocho and Viwandani). The primary goal of the NUHDSS was to provide a platform for investigating the long-term social, economic, and health consequences of urban residence and to serve as a principal research tool for intervention and impact evaluation studies focusing on the needs of the urban poor in SSA (62).

### Isiolo County

#### Identified data systems

##### Sources of data

In Isiolo County, there was only one source of ECD-related data, supported by a non-state actor focused on children’s nutrition. Additionally, no data were available on ECD platforms or sources for other components of the nurturing and care framework, such as responsive caregiving, safety, security, and ECD resourcing.

Standardized Monitoring and Assessment of Relief and Transition (SMART)—The standard SMART survey questionnaire was created using Kobo Toolbox and downloaded onto smartphones and tablets via the Open Data Kit mobile application. Daily plausibility checks were conducted to ensure the quality of anthropometric data and other datasets. Anthropometric data processing was performed using ENA software, while all other quantitative data were analyzed with MS Excel and SPSS (Version 20). The primary aim of the survey was to determine the prevalence of malnutrition among children aged 6–59 months and women of reproductive age (15–49 years) in Isiolo County (63).

### Homa Bay County

#### Identified data systems

##### Sources of data

In Homa Bay County, data on children’s health and nutrition were collected from both manual and digital sources. The results indicated that the county utilizes Maternal and Child Health (MCH) booklets, which are provided by the MoH at the national level and are widely used across Kenya. The digital sources included iCCM and the Pediatric Index Testing Cascade in Homa Bay, Kenya.

Integrated Community Case Management (iCCM)—Kenya’s primary health care system aims to provide essential health care based on practical, scientifically sound, and socially acceptable methods and technologies, made universally accessible to individuals and families in the community through their full participation, and at a cost that the community and country can afford (65). The iCCM services are built upon a functional community health system, which in turn leverages and links with the network of primary healthcare facilities (59).

Pediatric Index Testing Cascade in Homa Bay, Kenya—The Pediatric-focused Accelerated Case-finding Effort (PACE) initiative was introduced in March 2020 in 30 facilities across eight sub-counties of Homa Bay, Kenya, to improve HIV case identification in children (66).

### Stakeholders involved in ECD data systems/platforms

The findings reveal a diverse range of stakeholders across various data platforms in Kenya and other countries in SSA. These stakeholders can be categorized into the following groups: government institutions, NGOs/non-state actors, healthcare and community-based workers, and academia and research institutions.

Government institutions: Ministry of Health (MoH), Ministry of Education (MoE), Kenya National Bureau of Statistics (KNBS), National Council for Children’s Services (NCCS).

NGOs/Non-State Actors: UNICEF, WHO, World Bank, USAID, LEGO Foundation, PATH, FHI 360, Save the Children, World Vision.

Healthcare and community-based workers: Community Health Workers/Promoters (CHW/Ps), Nurses, Clinicians, Health Facility Staff & Administrators.

Academic & Research Institutions; Universities (e.g., Moi University, Kenyatta University, University of Nebraska Medical Center) and Research Partners (e.g., Ifakara Health Institute, London School of Hygiene & Tropical Medicine, APHRC).

### Challenges experienced by the ECD data sources and platforms

Despite advances in the digitization of ECD data across SSA, most data sources and platforms face several challenges, including infrastructure and technical limitations, data quality and completeness issues, human resource and capacity gaps, systemic fragmentation and policy disconnect, and methodological and reporting weaknesses.

Many health and education systems struggle with unreliable electricity, poor internet connectivity, and outdated technology. In some regions, health workers rely on solar power banks to charge their mobile devices (39), while others utilize networks from neighboring countries due to local connectivity gaps. Disease-specific electronic health record (EHR) systems, such as those for HIV and TB, often operate in isolation, lacking interoperability with broader platforms like DHIS2 (71). Additionally, software modifications are frequently restricted to developer teams, creating bottlenecks in system adaptability (71).

Missing, inconsistent, and duplicated data can significantly impact health and education management systems. In Kenya, maternal health data in DHIS2 is often incomplete because many deliveries occur outside formal health facilities (53). Clinics sometimes record patient information on loose sheets due to missing registers, leading to error-prone data (52). Surveys also face biases, such as variations affecting malaria net usage statistics (20), while self-reported data from household surveys may be affected by recall and social desirability biases (48).

High staff turnover and insufficient training hinder effective data management. New health workers often lack familiarity with digital systems, leading to incorrect data entry (52). Clinicians frequently delegate data tasks to clerks, reducing engagement with EHRs (71). In education, many countries rely on manual data collection, which has limited capacity to analyze and utilize information for policy decisions (48).

Health and education data systems are often siloed, with poor integration across sectors. ECD indicators, for example, are scattered across health, nutrition, and education databases, making holistic analysis difficult (48). Political and bureaucratic delays further stall progress, Tanzania’s ECD dashboard faced approval bottlenecks, which slowed data integration (43). Additionally, national policies, such as iCCM, often lack localized implementation strategies (65).

Many systems prioritize quantitative data while neglecting qualitative insights, leading to gaps in understanding vulnerable populations (47). Additionally, platforms that rely on SDG monitoring frameworks are often misaligned with national priorities, leaving countries struggling to meet reporting requirements (45). In establishing the ECD platform, for the Tanzanian ECD dashboard, the challenges noted were delays in securing approval for the integration of data from the sector-specific information management system (organizational policy, political will). This includes aligning the requirements with the information already collected from other sources and ensuring the reliability of the data gathered from various sources (43).

### Lessons learnt on the ECD data sources and platforms

The findings of this scoping review highlight several recommendations that could be integrated into the proposed ECD data visualization platform to address potential challenges, improve usability, and enhance overall functionality. Such recommendations include balancing flexibility and functionality during platform design, ensuring data quality, engaging stakeholders for buy-in and ownership, and multisectoral integration (interoperability).

A successful ECD data visualization platform should be capable of accommodating diverse data sources while remaining intuitive and user-friendly. Lessons from DHIS2 implementations and electronic health records (71) highlight:

- Offline functionality: Network disruptions are common; an offline mode ensures uninterrupted data entry (21).
- Integration with existing systems: DHIS2 compatibility can reduce training burdens, but overextension should be avoided (27).

Therefore, the ECD data visualization platform prototype should be designed with offline capabilities and tested for usability in pilot counties before being scaled.

Data quality is crucial to ensure that the collected data accurately reflects the current state of ECD indicators, enabling informed and effective decision-making. Based on this review’s findings, quality checks could be improved by adding automated validation (28) and triangulating existing external data sources to verify accuracy (22). Furthermore, a feedback loop could be implemented, where the data quality audit guides ongoing training and continuous platform improvement efforts.

To improve stakeholders’ engagement, the data visualization project should adopt a participatory design approach and collaborate with immediate users of ECD data (using both top-down and bottom-up rollout approaches) (46). Additionally, there should be sustainable support structures, such as help desks to resolve technical issues promptly and a community of practice (CoP) for peer learning among the three piloting counties. Furthermore, to ensure the effective piloting and assessment of the platform’s feasibility and acceptability, targeted sensitization and advocacy for its use should be implemented.

Recognizing that ECD is multisectoral, there is a need to break down sectoral silos and foster greater integration and collaboration across multiple sectors. The data visualization platform could leverage the drafted Kenya National ECD Policy 2022’s M&E framework to standardize indicators across various sectors. In addition, a multisectoral coordination team could also be established in each county to harmonize the ECD indicators across sectors.

## Discussion

This review aimed to map existing data systems and platforms providing ECD data across SSA, with a special focus on Kenya. It further details indicators within the NCF, the stakeholders involved, as well as the strengths and challenges. The review revealed a siloed ecosystem of systems, ranging from regional dashboards like UNICEF’s, the National Evaluation Platform (Malawi, Mali, Mozambique, and Tanzania), NeoTree (Malawi), the Early Childhood Development Dashboard (Tanzania), and Ecdinfohub.org (South Africa). In Kenya, child health digital platforms include the Antenatal, Intrapartum, and Postnatal Care COVID-19 Surveillance Study (ANCOV), the Electronic Health Record System (Afya EHMS), the Demographics Kenya Dashboard, the Kenya County Health Fact Sheets dashboard, the Integrated Community Case Management (iCCM) platform, Kenya Health Management Information System (KHMIS), District Health Information Software 2 (DHIS2), and the electronic Community Health Information System (eCHIS). In education, EMIS (now KEMIS), and in social protection, the Child Protection Management Information System (CPIMS). Although this diversity indicates a solid foundation of data collection, our analysis uncovers critical gaps in integration, accessibility, and utility that ultimately hinder effective, data-driven decision-making for young children.

The platforms identified can be characterized along three dimensions: (1) By primary data source the majority rely on facility-based data (e.g., DHIS2, EMR, iCCM), while a smaller subset draw from population-based household surveys (DHS, MICS), and a few integrate both; (2) By governance most platforms are government-led (through Ministries of Health or Education), though a significant proportion are managed or co-funded by multilateral or NGO partners (UNICEF, WHO, USAID); (3) By operational status several platforms appear inactive or infrequently updated (e.g., KenInfo last updated November 2013; Wazi Map Kenya relying on 2009 census data), raising systemic questions about data currency and long-term sustainability.

A critical but underexplored dimension of the ECD data landscape is data use: not merely whether data are collected, but whether they are applied to drive decisions. Several reviewed systems, particularly EMIS and DHIS2, collect substantial data but are noted in the literature as infrequently used for subnational planning decisions (47, 48). The reviewed literature identified limited documented instances of ECD platform data directly driving specific policy or resource allocation decisions at the county level in Kenya. The absence of such data-to-decision pathways should be treated as a significant gap: a future integrated ECD platform must embed feedback mechanisms that create institutional accountability for data use, not merely for data capture.

Digital health data has been promoted in Africa to improve healthcare quality and support decision-making within the healthcare system and beyond. It enables efficient, timely entry and storage of relevant health data, ensuring real-time monitoring and analysis that support evidence-based decisions. HDD gives health professionals and administrators easier access to both historical and current patient data, helping them analyze this information and make more informed choices. This data-driven approach assists health professionals in selecting appropriate treatments and helps policymakers create effective health policies (72). The review confirms a significant shift towards digitization, with most platforms utilizing electronic data from collection to reporting. This transition promises greater efficiency, timelier data, and enhanced analysis potential (72). However, this progress is markedly uneven across sectors and geographic contexts: counties with weaker digital infrastructure, predominantly remote and ASAL settings such as Isiolo are systematically underrepresented in digital ECD data ecosystems, creating a paradox in which children most at risk of developmental adversity are also least visible in national platforms. In sectors like education, partial digitization remains a barrier, impeding data quality and the timeliness of its use.

### Why ECD data systems remain fragmented

ECD Data fragmentation could be attributed to five key factors. The first is governance, ECD is constitutionally and administratively a shared mandate: in Kenya, health and pre-primary education are devolved functions while civil registration, national statistics and social protection remain national, so no single ministry or county department holds the authority to compel cross-sectoral reporting on children under five. Devolution reorganised accountability for service delivery without correspondingly reorganizing information flows, a mismatch documented in comparative work on decentralization and health system governance (73). In the absence of a designated data custodian for ECD, each sector optimizes its own system for its own reporting obligations, and integration becomes nobody’s key performance indicator.

The second is financing, as the review found that ECD budget lines are indicator- or sector-specific rather than child-centred, and information systems are typically funded as components of programme grants rather than as durable public infrastructure. This produces a characteristic life cycle in which a platform is built to a donor specification, performs during the grant period, and then decays: KenInfo, last updated in 2013, and Wazi Map, still resting on 2009 census data. Sustainability failure here is fiscal before it is technical. The third is capacity, and it operates at two levels: at the individual level, the review found recurrent shortages of personnel able to maintain, query and interpret the systems. At the institutional level, proprietary software creates a structural dependency in which only the original development team can modify the platform, so that departure of that team effectively ends the system’s life.

The fourth is digital infrastructure, whose effects are strongly regressive. Counties with weaker connectivity and power reliability, predominantly the arid and semi-arid frontier counties such as Isiolo, are systematically underrepresented in national digital ECD data. The result is that children at greatest risk of developmental adversity are the least legible to the systems that allocate resources, and any national platform that does not explicitly correct for this will inherit and amplify inequity. The fifth is political economy: notably, a ministry that shares its data also shares the ability of others to scrutinize its performance, and interoperability redistributes not only information but influence. Where indicator definitions differ across sectors, harmonisation implies that one sector’s definition prevails, which is a negotiation rather than a technical exercise. This helps explain a finding that recurs in the DHIS2 implementation literature as much as in the present review: technical interoperability is achieved far more often than institutional interoperability (13, 14).

### Findings in relation to regional and global frameworks

Set against the wider evidence base, the picture reported here is consistent with and extends three established literatures. First, the DHIS2 literature repeatedly finds that implementation strength is uneven across the data value chain, with substantial progress in capture and reporting but persistently limited routine use of the resulting data for subnational decision-making (15, 16). Second, the WHO Global Strategy on Digital Health 2020–2025 sets out enterprise architecture, data standards and interoperability as the foundations of national digital health systems (13). The systems mapped here largely predate or sit outside this architecture, which is why linkage failures, such as that between the NEP platform and DHIS2, recur. Third, comparative benchmarking through the Global Digital Health Monitor shows digital health maturity across the WHO African Region to be uneven rather than uniformly low, with standards and interoperability varying widely between countries and frequently lagging behind governance (12). Our county-level findings suggest that national maturity scores also conceal substantial within-country variation that matters greatly for ECD equity. This review adds to that literature by showing that the deficit is not evenly distributed across the Nurturing Care Framework: responsive caregiving and safety and security are not merely under-digitized; they are largely unmeasured, and no amount of interoperability between existing systems will generate indicators that no system currently collects. Notably, UNICEF in collaboration with Countdown to 2030, has compiled a few ECD related indicators across countries with African regions aged in 2025. However, this compilation focuses on national/country profiles with no aggregation at the county level.

### Implications for universal health coverage, the SDGs and health information systems

These findings carry implications beyond the ECD sector. For universal health coverage, monitoring depends on the ability to identify who is being reached and who is being missed; where children in ASAL counties are systematically underrepresented in routine digital systems, UHC coverage estimates for this age group carry an equity bias that is invisible in the reported figures. For the Sustainable Development Goals, indicator 4.2.1 on developmental on-track status is precisely the kind of cross-domain measure that this review finds no routine system to produce; SDG 4.2.1 reporting in the region therefore continues to depend on periodic surveys, with the reporting lags and limited subnational granularity that entails, while indicators 16.9.1 on birth registration and 2.2.1 and 2.2.2 on stunting, wasting and overweight sit in separate systems that are not linked at child level. For health information systems more broadly, the ECD case is a demanding test: it requires linkage not only across facilities within the health sector but across health, education, civil registration and child protection, under different ministries and different legal bases for data sharing. A system architecture that can carry ECD indicators can carry most other cross-sectoral agendas, which is an argument for treating ECD integration as a strategic priority rather than a niche one.

### Implications for the design of an integrated ECD data platform

Four design commitments follow from the evidence. The first is a minimum ECD indicator package. Rather than attempting to federate every indicator held in every system, national stakeholders should agree a parsimonious core set spanning all five NCF domains in the order of 20 to 30 indicators, each with an agreed numerator, denominator, disaggregation and reporting period drawn where possible from indicators already collected, and explicitly commissioning new collection for responsive caregiving, where this review found almost no routine measurement. Anchoring the package to SDG 4.2.1, the NCF and existing county reporting obligations would maximize both comparability and the likelihood of institutional uptake.

The second is interoperability by standard. The platform should exchange data using recognized specifications rather than bespoke point-to-point integrations: HL7 FHIR resources for individual-level clinical and immunization data, the OpenHIE architecture for the component pattern of client, facility, product and terminology registries linked through an interoperability layer, and DHIS2 aggregate exchange formats for routine indicator flows (13, 14). The practical consequence is that the ECD platform should function as a consumer and presentation layer over existing systems, not as a new system of record; this both avoids duplicate data entry, which the review identifies as a driver of poor data quality, and prevents the platform from becoming another silo. The third is metadata governance; fragmentation persists in part because the same indicator name denotes different things in different systems. A shared metadata registry defining each indicator, its permissible disaggregation, with a policy, is a precondition for meaningful integration.

The fourth is institutional embedding of data use. Given the recurrent finding that data are captured more reliably than they are used, the platform should be tied to specific decision moments: county ECD scorecards timed to County Integrated Development Plan reviews, and programme-based budget cycles, with a named accountable office in each county and a standing reporting line to the national ECD coordination structures could support uptake and sustainability.

The discussion below delves into key findings, including strengths, gaps, and challenges of the existing platforms in general, as well as those specific to ECD data, where applicable. The lessons and later the recommendations are useful information on best practices for developing effective and sustainable data platforms, particularly a centralized ECD data platform for which the review was conducted.

### Strengths of the systems

The review revealed several data systems in the region, some covering the entire SSA, while others covered only a few countries or a single country. The findings reveal several data systems and dashboards in the health sector, as well as a few in education. Notably, only three multisectoral dashboards (the UNICEF dashboard, Ecdinfohub.org, and the Tanzania ECD dashboard) report on indicators across all NCF domains. Further, while the platforms tended to cover data for the general population, with children’s data aggregated with the rest of the population data, a few platforms focused exclusively on ECD data and were multisectoral. The Tanzania ECD Data Dashboard is a good example of a multisectoral platform. Whereas having multiple data systems that are sector-specific may be convenient and allow sectors to manage their data independently, the absence of an integrated (multisectoral) ECD-specific platform means that policy-makers, service delivery personnel, and other data users needing data on children will have to search several sources where they are scattered, and sometimes not disaggregated by age. This may make it tedious for decision-makers to use data to plan, program, and allocate resources, ultimately affecting service delivery at the regional, national, or subnational levels.

An interesting finding was that a lot of the platforms reviewed were, to a good extent, digitized (using electronic data) from the point of collection to reporting. This aligns with the global shift to electronic systems; however, the results showed that, for example, in the education sector, the digitization of ECD data had Enhancing ECD data digitization particularly in the education and social protection sectors requires a phased approach: standardized digital ECD templates within existing platforms (e.g., a dedicated ECD module within KEMIS or CPIMS) in the short term; mobile-first tools with offline functionality (similar to CommCare and Kobo Toolbox models) (39) for remote settings in the medium term; and, in the long term, a national ECD data standard aligned with the NCF and mandated in policy, making digitization a systemic requirement, yet to be achieved, impeding data quality and the timeliness of its use.

Policy Relevance and Subnational Integration. The platforms identified in this review notably DHIS2, NEMIS/KEMIS, and the Child Protection Management Information System, already feed into existing government reporting cycles. County Integrated Development Plans (CIDPs), Annual Development Plans, and County Programme-Based Budgets are the primary vehicles through which county governments translate ECD policy into funding decisions. A well-designed integrated ECD platform could serve as a data hub producing quarterly or annual ECD scorecards at county level, directly informing CIDP reviews and budget allocation. Key stakeholders who should champion such a platform include the Ministry of Health (DHIS2 gateway), the Ministry of Education (KEMIS integration), the KNBS (statistical standards), and UNICEF/WHO (technical support). Explicit stakeholder mapping and institutional buy-in from these actors should be prerequisites for platform development.

What also stands out is that having sufficient data about education, health, or other services can enable LMICs to make better decisions. For instance, when EMIS data are gathered reliably, central and local government entities can identify shortages of qualified teachers and which schools require more infrastructure, such as classroom construction. Many countries collect data and upload it to the system. However, the reviewed literature identifies limited documented instances of these statistics driving specific programme or budget decisions at the county level in Kenya, suggesting a persistent disconnect between data availability and data use, but few use the statistics for decision-making. Systems should be in place to prompt the use of EMIS data to inform decision-making. Such systems could be like the WB projects that used EMIS, thereby prompting governments to continuously carry out national learning assessments (71).

### Challenges/gaps in the systems

The review also reveals capacity gaps in both resources and human resources. Apart from the few systems managed by UNICEF, WHO, or other relevant organizations. Many of the data systems were managed within government frameworks and involved personnel at different levels of the data pathway. A lack of infrastructure (hardware outdated computers unable to run current software versions) and proprietary software (where only the original developer team can make modifications, creating structural fragility once that support ends), as well as the skills to use and maintain the platforms, was one of the challenges highlighted. For instance, if only the developer team could make software modifications to the system, the platform was likely to become extinct as soon as the developers left (64). The sustainability of the platform, therefore, requires capacity and a plan to continue training personnel on its management and use, as well as ensuring that the necessary infrastructure, including reliable internet and power supply, is available, or a budget is allocated for maintaining these. In remote and resource-limited settings, it would therefore be useful to have data systems that allow data to be updated offline and sync the information as soon as the internet is available.

Data quality is a common challenge with routine health data systems. For example, in South Africa, the quality of health data on the platforms varied, with the most significant problems being inconsistent recording of folder numbers and missing patient information (37). In DHS in Kenya, systematic non-representativeness, including the delayed start of ANC care attendance, as well as the significant proportion of maternal events, including deliveries, that occur outside health facilities, undermines data quality, accuracy, and completeness (53). Missing data was also a problem with other data systems; for example, the DHS, which lacked data for some East African countries (74). Missingness, such as for spatial data, where specific locations lacked data on anaemia, was resolved using interpolation; however, this addresses completeness but undermines precision. Furthermore, issues with limited interoperability between data systems usually hamper the utilization of data between data systems, as was exhibited by the NEP data platform, which could not easily be linked with the DHIS2 because of the different data analysis programs that were used (26). Moreover, large data systems often utilize tools that enable the pre-calculated indicators from various data sources to be consolidated into a single dataset. This means that users cannot manipulate data within the system (e.g., to modify the indicator definition). Such frameworks include the Health Metrics Network supported by the World Health Organization, and software (e.g., DevInfo supported by UNICEF) (40). This was also the challenge with the incomplete direct integration of Electronic Medical Records EMRs with DHIS2 for routine HIV data in Kenya (58). It is therefore essential that data systems are designed with formats and software that are compatible with local infrastructure, utilizing flexible software systems that facilitate communication across different languages and literacy levels. This approach also involves conducting iterative, participatory design with individuals who possess in-depth knowledge of local clinical and cultural norms.

In summary, several data systems/platforms in SSA, including those in Kenya, provide for ECD data. These are usually sector-specific with limited interoperability across sectors, hence the need for a system to enhance integration. Further, a few challenges with these platforms have been reported, including infrastructure and technical limitations, data quality and completeness issues, human resource and capacity gaps, systemic fragmentation, policy disconnects, and methodological and reporting weaknesses. Additionally, there is a significant lack of aggregated information or data on how children thrive within the nurturing care framework (NCF) domains. Findings from this review indicate that across sub-Saharan Africa, there are limited comprehensive, real-time, and actionable data sources, hindering decision-makers’ ability to implement timely, evidence-based interventions that could prevent developmental delays in the short and long term. This, therefore, calls for the development of a multisectoral (centralized) platform that pools data from multiple systems into a single platform for holistic visualization and reporting.

### Strengths and limitations

This review has four principal strengths. It is, to our knowledge, the first systematic mapping of ECD and ECD-related data systems across sub-Saharan Africa organized explicitly around the five domains of the Nurturing Care Framework, which allows the framework’s operational feasibility of existing tools to be mapped into the NCF as. Second, the inclusion of grey literature, government reports, platform documentation and county-level assessments captured a substantial number of operational systems that are not represented in the indexed peer-reviewed literature and would have been invisible to a conventional database-only search. Third, the nested design, moving from regional to national to county level, allowed profiling of three contrasting Kenyan counties and surfaced the sub-national infrastructure inequities that national-level analysis conceals. Fourth, the review was conducted against a registered protocol and reported in accordance with PRISMA-ScR, with the full checklist provided as Supplementary file A.

We recognize the limitations of scoping reviews and other forms of literature reviews; the information obtained may not be exhaustive, as not all information is always documented. There may be additional data systems and information about the identified systems that are not published; therefore, additional studies involving surveys of systems are likely to yield more information, enriching findings from literature.

Four additional limitations should be noted. First, the lower temporal boundary of August 2010, while grounded in Kenya’s constitutional devolution context, may have excluded pre-2010 ECD data systems in SSA countries where no equivalent governance transition occurred in that year. Second, the language restriction revised to include English, Kiswahili and French may still exclude evidence from Portuguese- and Arabic-speaking SSA settings, potentially affecting the comprehensiveness of SSA-wide findings. Third, publication and reporting bias is likely to operate asymmetrically: better-resourced and donor-supported platforms are more likely to be documented and therefore more likely to have been captured here, while functioning county-level systems that are undocumented, or documented only in internal records, will have been missed. Relatedly, because dual screening at the title and abstract stage was applied to a 10% sample rather than to the full record set, no chance-corrected measure of screening agreement can be reported. Fourth, the absence of a data-quality appraisal, the rapid evolution of digital platforms and the cross-sectional nature of the evidence may also affect the validity of these findings; the operational status recorded here represents a cross-section at the point of searching, and attributes such as update frequency and interoperability will have changed since.

## Conclusion and recommendations

Data systems are crucial for enhancing decision-making and delivering effective, equitable services, including those for children. This scoping review has identified several data systems that offer indicators for children, varying in geographical scope, reported information, and function. Overall, these systems hold valuable information on ECD across the nurturing care domains. However, they are primarily designed for the general population, operate in silos within specific sectors, and differ substantially in scope and capabilities. Consequently, a significant gap remains: no single platform in Kenya or elsewhere in SSA provides a comprehensive view of a child’s wellbeing across all NCF domains. The fragmentation of data across multiple systems creates a significant obstacle for policymakers and practitioners, underscoring the urgent need for a centralized ECD data platform. Such a platform would not replace existing systems but would integrate and standardize their indicators, enabling holistic visualization and empowering stakeholders to make truly coordinated, evidence-based decisions for children.

Despite these limitations, we propose several recommendations to guide the development of an effective and sustainable centralized ECD platform. Adopting an integrated, interoperable design to strategically position and technically design the new platform for seamless alignment with existing government and partner systems (e.g., DHIS2, KEMIS, CPIMS). Priority should be given to using flexible, interoperable software standards that facilitate data exchange and avoid the pitfalls of isolated, incompatible systems. Additionally, potential challenges should be addressed, including continuous training and skill transfer to local personnel to ensure long-term platform management and sustainability, and reducing reliance on external developers.

We therefore make five specific recommendations. First, national and county governments in SSA should adopt an interoperable national ECD data platform that integrates data from the existing systems, rather than replacing them, using recognized interoperability standards and aggregate exchange for routine indicator flows. Second, ministries responsible for ECD should agree and share a minimum ECD indicator package of 20 to 30 indicators spanning all five NCF domains, each with a defined numerator, denominator, disaggregation and reporting cadence, and should commission new routine collection for responsive caregiving, which this review found to be the least measured domain in the region. Third, a single institutional custodian for ECD data should be designated in each country, with a corresponding accountable office at the county level, since the absence of a data owner is a primary structural cause of the fragmentation documented in this review. Fourth, a shared metadata registry with versioning, named indicator custodians and published last-updated timestamps should be established, so that data currency is visible to users. Fifth, platform investment should be costed, including hosting, continuous personnel training and periodic revision, with offline-capable, mobile-first data capture specified. Implementation should be sequenced, beginning with an ECD module within existing platforms in the short term, mobile-first offline tools for remote settings in the medium term, and a policy-mandated national ECD data standard aligned to the NCF in the longer term.

## Data Availability

The original contributions presented in the study are included in the article/Supplementary material, further inquiries can be directed to the corresponding author.

## References

[1] Grantham-McGregor S Cheung YB Cueto S Glewwe P Richter L Strupp B. Developmental potential in the first 5 years for children in developing countries. Lancet. (2007) 369:60–70. doi: 10.1016/S0140-6736(07)60032-4, 17208643 PMC2270351

[2] Gil JD Ewerling F Ferreira LZ Barros AJD. Early childhood suspected developmental delay in 63 low- and middle-income countries: large within- and between-country inequalities documented using national health surveys. J Glob Health. (2020) 10:010427. doi: 10.7189/jogh.10.010427, 32566165 PMC7295453

[3] APHRC CoG, a.B.W.P. Making data systems work for counties: an assessment of data systems to support Investments in Children and Youth in counties in Kenya (2018). Available online at: https://aphrc.org/publication/making-data-systems-work-for-counties/

[4] Harvard School of Public Health SBI, G.C.C. One Third of Children in Low- and Middle-Income Countries Fail to Reach Developmental Milestones. Cambridge: The Harvard Gazatte (2016).

[5] UNICEF. The formative years: UNICEF’S work on measuring ECD. 8–11 (2014). Available online at: https://data.unicef.org/resources/the-formative-years-unicefs-work-on-measuring-ecd/

[6] UNICEF. In Homa Bay, teenage girls face high risk of HIV and unintended pregnancy gender inequality, difficulties in accessing services and poverty are fuelling high rates of teenage pregnancies and HIV in Homa Bay (2020). Available online at: https://www.unicef.org/kenya/stories/homa-bay-teenage-girls-face-high-risk-hiv-and-unintended-pregnancy

[7] WHO. Nurturing Care for Early Childhood Development. Geneva: WHO (2020).

[8] Countdown to 2030 Collaboration. Countdown to 2030: tracking progress towards universal coverage for reproductive, maternal, newborn, and child health. Lancet. (2018) 391:1538–1548. doi: 10.1016/S0140-6736(18)30104-1, 29395268

[9] Black MM Walker SP Fernald LCH Andersen CT DiGirolamo A Lu C . Early childhood development coming of age: science through the life course. Lancet. (2017) 389:77–90. doi: 10.1016/S0140-6736(16)31389-7, 27717614 PMC5884058

[10] Jeong J Bliznashka L Sullivan E Hentschel E Jeon Y Strong KL . Measurement tools and indicators for assessing nurturing care for early childhood development: a scoping review. PLOS Glob Public Health. (2022) 2:e0000373. doi: 10.1371/journal.pgph.0000373, 36962242 PMC10021181

[11] WHO. Global Strategy on digital Health 2020–2025. Geneva: World Health Organization (2021).

[12] WHO. Global Digital Health Monitor. Geneva: World Health Organization (2023).

[13] Dehnavieh R Haghdoost AA Khosravi A Hoseinabadi F Rahimi H Poursheikhali A . The district health information system (DHIS2): a literature review and meta-synthesis of its strengths and operational challenges based on the experiences of 11 countries. Health Inf Manag. (2019) 48:62–75. doi: 10.1177/1833358318777713, 29898604

[14] Byrne E Sæbø JI. Routine use of DHIS2 data: a scoping review. BMC Health Serv Res. (2022) 22:1234. doi: 10.1186/s12913-022-08598-8, 36203141 PMC9535952

[15] Levac D Colquhoun H O'Brien KK. Scoping studies: advancing the methodology. Implement Sci. (2010) 5:69. doi: 10.1186/1748-5908-5-69, 20854677 PMC2954944

[16] Arksey H O'Malley L. Scoping studies: towards a methodological framework. Int J Soc Res Methodol. (2005) 8:19–32. doi: 10.1080/1364557032000119616

[17] Tricco AC Lillie E Zarin W O'Brien KK Colquhoun H Levac D . PRISMA extension for scoping reviews (PRISMA-ScR): checklist and explanation. Ann Intern Med. (2018) 169:467–73. doi: 10.7326/M18-0850, 30178033

[18] Barroso J Gollop CJ Sandelowski M Meynell J Pearce PF Collins LJ. The challenges of searching for and retrieving qualitative studies. West J Nurs Res. (2003) 25:153–78. doi: 10.1177/0193945902250034, 12666641

[19] McHugh ML. Interrater reliability: the kappa statistic. Biochem Med (Zagreb). (2012) 22:276–82. doi: 10.11613/BM.2012.031, 23092060 PMC3900052

[20] Ng M Ellicott Colson K Fullman N Dwyer-Lindgren L Achoki T Schneider MT . Assessing the contribution of malaria vector control and other maternal and child health interventions in reducing all-cause under-five mortality in Zambia, 1990-2010. Am J Trop Med Hyg. (1990) 97:58–64. doi: 10.4269/ajtmh.15-0315, 26880778 PMC5619928

[21] Willcox M Moorthy A Mohan D Romano K Hutchful D Mehl G . Mobile Technology for Community Health in Ghana: is maternal messaging and provider use of technology cost-effective in improving maternal and child health outcomes at scale? J Med Internet Res. (2019) 21:e11268. doi: 10.2196/11268, 30758296 PMC6391645

[22] Ahinkorah BO Budu E Seidu AA Agbaglo E Adu C Ameyaw EK . Barriers to healthcare access and healthcare seeking for childhood illnesses among childbearing women in sub-Saharan Africa: a multilevel modelling of demographic and health surveys. PLoS One. (2021) 16:e0244395. doi: 10.1371/journal.pone.0244395, 33556057 PMC7870045

[23] Akuze J Blencowe H Waiswa P Baschieri A Gordeev VS Kwesiga D . Randomised comparison of two household survey modules for measuring stillbirths and neonatal deaths in five countries: the every newborn-INDEPTH study. Lancet Glob Health. (2020) 8:e555–66. doi: 10.1016/S2214-109X(20)30044-9, 32199123

[24] Allorant A Fullman N Leslie HH Sarr M Gueye D Eliakimu E . A small area model to assess temporal trends and sub-national disparities in healthcare quality. Nat Commun. (2023) 14:4555. doi: 10.1038/s41467-023-40234-9, 37507373 PMC10382513

[25] Birhanie AL Tessema ZT Endalew B Tamirat KS. Under-five mortality and its associated factors in sub-Saharan Africa: a multilevel analysis of recent demographic and health surveys data based on Bayesian approach. BMC Pediatr. (2025) 25:103. doi: 10.1186/s12887-025-05454-6, 39923019 PMC11806815

[26] Ford ND Stein AD. Risk factors affecting child cognitive development: a summary of nutrition, environment, and maternal-child interaction indicators for sub-Saharan Africa. J Dev Orig Health Dis. (2016) 7:197–217. doi: 10.1017/S2040174415001427, 26358240 PMC4800975

[27] Hazel E Wilson E Anifalaje A Sawadogo-Lewis T Heidkamp R. Building integrated data systems for health and nutrition program evaluations: lessons learned from a multi-country implementation of a DHIS 2-based system. J Glob Health. (2018) 8:020307. doi: 10.7189/jogh.08.020307, 30356499 PMC6188163

[28] Shabani J Salim N Bohne C Day LT Kumalija C Makuwani AM . Neonatal indicator data in Tanzania District health information system: evaluation of availability and quality of selected newborn indicators, 2015-2022. BMC Pediatr. (2015) 23:658. doi: 10.1186/s12887-025-05417-x, 39849367 PMC11755859

[29] Unkels R. On People, data and Systems: Perspectives on Routine Health data Processing and its Digitalization in Tanzania. Stockholm, Sweden: Karolinska Institutet (2023).

[30] Molenaar J Kikula A Kionga Y Berenge HT Benova L van Olmen J . Data for whom? Experiences and perceptions of a perinatal eRegistry in two hospitals in Mtwara region, Tanzania. BMJ Glob Health. (2024) 9:e016765. doi: 10.1136/bmjgh-2024-016765, 39572051 PMC11580263

[31] Agyekum MW Yeboah SB Dzradosi C Ofosu-Ampong K Quaye MO Donkoh C . Rural-urban differentials in early childhood education and child development: evidence from multiple Indicator cluster survey (MICS) in Ghana. PLOS Glob Public Health. (2023) 3:e0002171. doi: 10.1371/journal.pgph.0002171, 37549109 PMC10406182

[32] Bago J-LO Moussa Akakpo K Lompo ML Souratié W d M Ouédraogo E. Early childhood education and child development: new evidence from Ghana. Children Youth Serv Rev. (2020) 108. doi: 10.1016/j.childyouth.2019.104620

[33] McCoy DC Peet ED Ezzati M Danaei G Black MM Sudfeld CR . Early childhood developmental status in low- and middle-income countries: national, regional, and global prevalence estimates using predictive Modeling. PLoS Med. (2016) 13:e1002034. doi: 10.1371/journal.pmed.1002034, 27270467 PMC4896459

[34] UNICEF. Country Profiles; Kenya; Key Demographic Indicators. New York: UNICEF (2026).

[35] Arhin K Acheampong K Frimpong AO. Benchmarking the performance of healthcare systems in the provision of childhood immunization services in sub-Saharan Africa from 2016 to 2019. Cogent Soc Sci. (2016) 11:2471562. doi: 10.1080/23311886.2025.2471562

[36] Crehan C Kesler E Nambiar B Dube Q Lufesi N Giaccone M . The NeoTree application: developing an integrated mHealth solution to improve quality of newborn care and survival in a district hospital in Malawi. BMJ Glob Health. (2019) 4:e000860. doi: 10.1136/bmjgh-2018-000860, 30713745 PMC6340059

[37] Huber AN Pascoe S Fox MP Murphy J Mphokojoe M Gorgens M . Leveraging routine data in impact evaluation: understanding data systems in primary healthcare prior to a matched cluster-randomised evaluation of adherence guidelines in South Africa. S Afr Med J. (2022) 112:819–27. doi: 10.7196/SAMJ.2022.v112i10.14909, 36472333

[38] Chi BH Vwalika B Killam WP Wamalume C Giganti MJ Mbewe R . Implementation of the Zambia electronic perinatal record system for comprehensive prenatal and delivery care. Int J Gynaecol Obstet. (2011) 113:131–6. doi: 10.1016/j.ijgo.2010.11.013, 21315347 PMC3071887

[39] Balla K Malm K Njie O Hounto Ogouyemi A Uhomoibhi P Poku-Awuku A . Introducing field digital data collection systems into seasonal malaria chemoprevention campaigns: opportunities for robust evidence development and national e-health strategies. BMJ Glob Health. (2022) 7. doi: 10.1136/bmjgh-2021-007899, 35296463 PMC8928254

[40] Diallo CO Schiøler KL Drabo KM Samuelsen H. Stakeholder’s perception of the health information system performance in Burkina Faso. Pan Afr Med J. (2023) 44:155. doi: 10.11604/pamj.2023.44.155.35294 PMC10349617 37455872

[41] UNICEF. The countdown profiles: a tool for action: the countdown RMNCAH county profiles present the latest evidence to assess progress in improving women’s, children’s, and adolescents’ health. (2026) Available online at: https://data.unicef.org/countdown-2030/

[42] UNICEF. Collecting Data on Early Childhood Development in Household Surveys. New York: UNICEF (2026).

[43] Elisaria E. Develop Indicators to be Included in the National Multisectoral ECD Scorecard. Edward Elgar Publishing, Cheltenham, United Kingdom: Ifakara Health Institute (2026).

[44] Yousuf Hussein S Swanepoel DW Mahomed F Biagio de Jager L. Community-based hearing screening for young children using an mHealth service-delivery model. Glob Health Action. (2018) 11:1467077. doi: 10.1080/16549716.2018.1467077, 29764328 PMC5954481

[45] Arnott A. Chapter 6: Priorities for Missing Data and SDG 4 for Countries in Africa. Cheltenham, United Kingdom: Edward Elgar Publishing (2024). p. 85–102.

[46] Benda N Dougherty K Gebremariam Gobezayehu A Cranmer JN Zawtha S Andreadis K . Designing electronic data capture systems for sustainability in low-resource settings: viewpoint with lessons learned from Ethiopia and Myanmar. JMIR Public Health Surveill. (2024) 10:e47703. doi: 10.2196/47703, 38345833 PMC10897790

[47] Hossain M. Large-scale data gathering: exploring World Bank’s influence on national learning assessments in LMICs. Int J Educ Dev. (2023) 102:102877. doi: 10.1016/j.ijedudev.2023.102877

[48] Wyk LCaCV. Efficiency and Effectiveness in Choosing and Using an EMIS. New York: UNICEF (2020).

[49] Aurino E Wolf S Tsinigo E. Household food insecurity and early childhood development: longitudinal evidence from Ghana. PLoS One. (2020) 15:e0230965. doi: 10.1371/journal.pone.0230965, 32243485 PMC7122750

[50] cdinfohub.org. Data and statistics on early childhood: overview of ECD in South Africa (2026). Available online at: https://ecdinfohub.org/data-statistics/

[51] Giese S Dawes A Biersteker L Girdwood E Henry J. Using data tools and systems to drive change in early childhood education for disadvantaged children in South Africa. Children (Basel). (2023) 10:1470. doi: 10.3390/children10091470, 37761431 PMC10529679

[52] CRS. Routine Data Quality Assessment Report for the Isiolo and Marsabit County Health System. Nairobi, Kenya: Catholic Relief Services (2022).

[53] Izulla P Wagai JN Akelo V Ombeva A Okeri E Onyango D . Vaccine safety surveillance in Kenya using GAIA standards: a feasibility assessment of existing national and subnational research and program systems. Vaccine. (2023) 41:5722–9. doi: 10.1016/j.vaccine.2023.07.063, 37550143

[54] Karimi J. Monitoring Progress in Implementation of ECD Policies, Kenya (2023). nurturing-care.org. Available online at: https://nurturing-care.org/wp-content/uploads/2023/12/kenya-monitoring.pdf

[55] Ndisha M Hassan AS Ngari F Munene E Gikura M Kimutai K . Leveraging electronic medical records for HIV testing, care, and treatment programming in Kenya-the national data warehouse project. BMC Med Inform Decis Mak. (2023) 23:183. doi: 10.1186/s12911-023-02265-6, 37715195 PMC10503194

[56] Ngugi PN Gesicho MB Babic A Were MC. Assessment of HIV data reporting performance by facilities during EMR systems implementations in Kenya. Stud Health Technol Inform. (2020) 272:167–70. doi: 10.3233/SHTI200520, 32604627

[57] Muinga N Magare S Monda J Kamau O Houston S Fraser H . Implementing an open source electronic health record system in Kenyan health care facilities: case study. JMIR Med Inform. (2018) 6:e22. doi: 10.2196/medinform.8403, 29669709 PMC5932328

[58] Owuor JO. "Information platforms and electronic data services relevant to human nutrition in Kenya". In: Global Support Facility for the National Information Platforms for Nutrition Initiative. Montpellier: Agropolis International (2017)

[59] USAID Gap analysis and investment case for integrated community case management (iCCM) (2012). Available online at: https://www.scribd.com/document/863923634/ICCM-Gap-Analysis-Investment-Case-final-Report

[60] Kenya M. M.O.E: advance school census questionnaire and data capture app (2024). Available online at: https://www.knbs.or.ke/wp-content/uploads/2025/01/2024-National-School-Census-Pilot-Report.pdf

[61] Incubator G.D. Strengthening early childhood development (ECD) delivery in Kenyan counties. In., vol. 2025 (2024). Available online at: https://globaldevincubator.org/strengthening-early-childhood-development-ecd-delivery-in-kenyan-counties/

[62] Beguy D Elung'ata P Mberu B Oduor C Wamukoya M Nganyi B . Health & Demographic Surveillance System Profile: the Nairobi urban health and demographic surveillance system (NUHDSS). Int J Epidemiol. (2015) 44:462–71. doi: 10.1093/ije/dyu251, 25596586

[63] USAID. Isiolo County Integrated Smart Survey Report. Washington: USAID (2024).

[64] Oyungu E Roose A Ombitsa AR Vreeman RC McHenry MS. Child development monitoring in well-baby clinics in Kenya. Int J MCH AIDS. (2021) 10:128–33. doi: 10.21106/ijma.473, 34026324 PMC8130212

[65] Onono M Abdi M Opondo I Okung'u J Asadhi E Nyamai R . Using the RE-AIM framework to evaluate the implementation of integrated community case management in Kenya. Acta Paediatr. (2018) 107:53–62. doi: 10.1111/apa.14662, 30570791

[66] Gill MM Musee P Herrera N Siamba S Mayi A. Identifying Gaps in the Pediatric Index Testing Cascade in Homa Bay, Kenya. (2023)

[67] County, M.H. Homa Bay County Nutritional Capacity Assessment Report (2017). Available online at: https://nutrition.health.go.ke/reports-capacity-assessment-reports/capacity-assessment-reports/capacity-assessment-reports-2/

[68] Hailu BA. Mapping, trends, and factors associated with anemia among children aged under 5 y in East Africa. Nutrition. (2023) 116:112202. doi: 10.1016/j.nut.2023.112202, 37832168

[69] Gao Y Zhang L Kc A Wang Y Zou S Chen C . Housing environment and early childhood development in sub-Saharan Africa: a cross-sectional analysis. PLoS Med. (2021) 18:e1003578. doi: 10.1371/journal.pmed.1003578, 33872322 PMC8092764

[70] KNBS. Demographic Health Survey. Nairobi: KNBS (2022).

[71] Kimuyu NFWM. Strengthening data Systems within Learning Health Systems in Kenya: Stepping back to step Forward. Vrije Universiteit Amsterdam (2023).

[72] Serge B . Health data Digitalization in Africa: Unlocking the Potential. Brazzaville: WHO Regional Office for Africa (2024).

[73] McCollum R Limato R Otiso L Theobald S Taegtmeyer M. Health system governance following devolution: comparing experiences of decentralisation in Kenya and Indonesia. BMJ Glob Health. (2018) 3:e000939. doi: 10.1136/bmjgh-2018-000939, 30294460 PMC6169670

[74] Free C Phillips G Galli L Watson L Felix L Edwards P . The effectiveness of mobile-health technology-based health behaviour change or disease management interventions for health care consumers: a systematic review. PLoS Med. (2013) 10:e1001362. doi: 10.1371/journal.pmed.1001362, 23349621 PMC3548655

