# Supplementary material for: Mapping early childhood development data systems in Kenya and sub-Saharan Africa: a scoping review
Source: Front Public Health. 2026 Oct 22;14:1936644. doi: 10.3389/fpubh.2026.1936644 (PMC13736838; doi:10.3389/fpubh.2026.1936644)
Supplement: Supplementary file 1 [file Supplementary_file_1.docx]

**Supplementary File A**

**PRISMA-ScR Checklist — Preferred Reporting Items for Systematic Reviews and Meta-Analyses extension for Scoping Reviews**

*Manuscript: Mapping and profiling Early Childhood Development (ECD) and related data systems in Kenya and across Sub-Saharan Africa: a scoping review*

Checklist adapted from: Tricco AC, Lillie E, Zarin W, et al. PRISMA Extension for Scoping Reviews (PRISMA-ScR): Checklist and Explanation. Ann Intern Med. 2018;169(7):467–473. doi:10.7326/M18-0850.

| **SECTION** | **ITEM** | **PRISMA-ScR CHECKLIST ITEM** | **REPORTED ON** |
| --- | --- | --- | --- |
| **TITLE** | 1 | Identify the report as a scoping review in the title. | title |
| **ABSTRACT — Structured summary** | 2 | Structured abstract covering background, objectives, eligibility criteria, sources of evidence, charting methods, results, and conclusions linked to the review questions and objectives. |  |
| **INTRODUCTION — Rationale** | 3 | Describe the rationale for the review in the context of existing knowledge and explain why the questions suit a scoping review approach. | Introduction |
| **INTRODUCTION — Objectives** | 4 | Provide an explicit statement of the questions and objectives addressed, including key elements (population, concept, context) and other elements used to frame the review. | Abstract; Identification of the research question, incl. Table 1: 3WH framework) |
| **METHODS — Protocol and registration** | 5 | State whether a review protocol exists and where it can be accessed; if available, give registration information including the registration number. | In the Methods |
| **METHODS — Eligibility criteria** | 6 | Specify the characteristics of sources of evidence used as eligibility criteria (e.g., years covered, language, publication status) with rationale. | Study selection: inclusion and exclusion criteria |
| **METHODS — Information sources** | 7 | Describe all information sources searched (databases with coverage dates; contact with authors to identify additional sources) and the date of the most recent search. | Identifying relevant studies |
| **METHODS — Search** | 8 | Present the full electronic search strategy for at least one database, including limits used, such that it could be repeated. | Table 2: search strategies per region |
| **METHODS — Selection of sources of evidence** | 9 | State the process for selecting sources of evidence (screening and eligibility) used in the review. | Study selection |
| **METHODS — Data charting process** | 10 | Describe the methods for charting data from the included sources (e.g., calibrated forms, independent or duplicate charting, processes for obtaining and confirming data from investigators). | Charting the data |
| **METHODS — Data items** | 11 | List and define all variables for which data were sought and any assumptions or simplifications made. | Charting the data |
| **METHODS — Critical appraisal of individual sources of evidence** | 12 (optional) | If done, describe the methods for appraising the included sources of evidence and how this information was used. | Not applicable |
| **METHODS — Synthesis of results** | 13 | Describe the methods used to handle and summarize the charted data. | Collating, summarizing, and reporting the results |
| **RESULTS — Selection of sources of evidence** | 14 | Give numbers of sources screened, assessed for eligibility, and included, with reasons for exclusion at each stage, ideally via a flow diagram. | Figure 1: PRISMA-ScR flow diagram |
| **RESULTS — Characteristics of sources of evidence** | 15 | For each included source, present the characteristics for which data were charted, with citations. | Results; system characteristics tables |
| **RESULTS — Critical appraisal within sources of evidence** | 16 (optional) | If done, present data on critical appraisal of the included sources. | Not applicable |
| **RESULTS — Results of individual sources of evidence** | 17 | For each included source, present the relevant data charted in relation to the review questions and objectives. | Results: SSA, Kenya, and county-level subsections by NCF domain |
| **RESULTS — Synthesis of results** | 18 | Summarize and/or present the charted results as they relate to the review questions and objectives. | Stakeholders; Challenges; Lessons learnt |
| **DISCUSSION — Summary of evidence** | 19 | Summarize the main results (including an overview of concepts, themes, and types of evidence available), link to the review questions and objectives, and consider relevance to key groups. | Discussion |
| **DISCUSSION — Limitations** | 20 | Discuss the limitations of the scoping review process. | Limitations |
| **DISCUSSION — Conclusions** | 21 | Provide a general interpretation of the results with respect to the review questions and objectives, and discuss potential implications and/or next steps. | Conclusion and Recommendations |
| **FUNDING** | 22 | Describe sources of funding for the included sources of evidence and for the scoping review, and the role of the funders. | Funding |
