# Supplementary material for: Mapping early childhood development data systems in Kenya and sub-Saharan Africa: a scoping review
Source: Front Public Health. 2026 Oct 22;14:1936644. doi: 10.3389/fpubh.2026.1936644 (PMC13736838; doi:10.3389/fpubh.2026.1936644)
Supplement: Supplementary file 2 [file Table_1.docx]

**Supplementary File D**

**Supplementary Table S1: Indicators from ECD data sources and platforms in sub-Saharan Africa**

Supplementary material accompanying: Mapping Early Childhood Development Data Systems in Kenya and Sub-Saharan Africa: A Scoping Review.

| **Data systems/platforms** | **Nurturing Care Framework** | **Domain/categories** | | **Indicators** |
| --- | --- | --- | --- | --- |
| The Zambia Electronic Perinatal Record System (ZEPRS) | Health | Current pregnancy | | Sample variables collected: Estimated due date (calculated from last menstrual period, fundal height, and/or ultrasound); singleton or multiple gestation; pregnancy complications; prior history of HIV testing; maternal immunizations received; use of insecticide-treated bed net. |
|  |  | Past obstetrical history | | Gravidity; parity; date and site of past delivery; mode of delivery; duration of labor; pregnancy complications (e.g., infection, eclampsia, hemorrhage); sex of infant; infant birth weight; infant's current vital status |
|  |  | Past medical history | | Review of symptoms; previous medical conditions, including hypertension, diabetes, asthma, heart disease, kidney disease, liver disease, thyroid disease, tuberculosis, HIV, and recent malaria; history of surgery, particularly pelvic surgery; drug allergies; past contraception use |
|  |  | Routine prenatal visit | | Estimated gestational age; fundal height measurement; fetal lie, presentation, and descent; fetal heart rate; blood pressure measurement; maternal weight; complete physical examination; HIV counseling and testing results (CD4 screening and result for those with HIV diagnosis); antiretroviral drug regimen prescribed; syphilis screening and test result; penicillin treatment administered; partner screening and treatment for HIV and syphilis; administration/dispensation of routinely used drugs in pregnancy (e.g. iron, folate, mebendazole, sulfadoxine/pyrimethamine); results from routinely performed laboratory tests (e.g. hemoglobin, urine analysis, malaria blood smear) |
|  |  | Labor and delivery | | Estimated gestational age; review of symptoms including contractions, fetal movement, vaginal bleeding, and ruptured membranes; fundal height measurement; fetal lie, presentation, and descent; blood pressure measurement; complete physical examination; fetal heart rate; vaginal examination, including cervical dilation, effacement, and position; clinical pelvimetry measurements; medications used to induce or augment labor; mode of delivery; complications of labor (e.g. chorioamnionitis, prenatal or postpartum hemorrhage, pre-eclampsia or eclampsia, dystocia); date and time of birth; infant sex; infant birth weight; Apgar scores at 1, 5, and 10 minutes; neonatal resuscitation measurement used; neonatal physical examination; infant dosing for HIV prophylaxis |
| Mobile Technology for Community Health (MOTECH) | Health | Economic costs were estimated from a program perspective. Health effects were modelled, including birth attendants, facility delivery, measles immunization, resources required, capital and recurrent expenditures for MOTECH, and the number of facilities or nurses (trained) | | |
| The Demographic and Health Survey (DHS) | Health | - Birth outcomes: Live births, stillbirths, miscarriages, and neonatal deaths. - Anaemia below 5 years preceding the birth interval, child sex, source of drinking water, type of fuel, toilet facility, Nutrition status: child stunting, wasting, weight status | | |
|  | Safety and security | - source of drinking water - type of fuel - toilet facility | | |
| Tanzania District Health Information System (DHIS2) | Health | - Health 20 indicators on neonatal health, i.e., Every Newborn Action Plan (ENAP) indicator (neonatal mortality, morbidity, breastfeeding, maternal indicators | | |
| Tanzania E-Registry | Health and nutrition | - Maternal and child health-related data. | | |
| Tanzanian Early Childhood Development Dashboard | Nutrition | - Proportion of children 6-23 months received Minimum Acceptable Diet - Proportion of parents/caregivers of children 0-23 months counselled on IYCF at the health facility - Proportion of parents/caregivers of children 0-23 months counselled on IYCF at the community level - Proportion of children 0 - 5 months exclusively breastfed survey - Proportion of children 0 - 23 months breastfed within one hour after birth, survey - Proportion of children 0 -59 months who are underweight, survey - Proportion of children 0 -59 months who are underweight, according to routine - Proportion of children 0 - 59 months with Acute Malnutrition survey - Proportion of children 0 - 59 months with Acute Malnutrition routine | | |
|  | Health | - Proportion of newborn deaths that occur within the first 28 days of life per 1000 live births- - Proportion of newborn deaths that occur per 1000 live births - Proportion of infants dying within the first year of life per 1000 live births, survey - Proportion of children under five years who are stunted, survey - Proportion of children under five years who are stunted routine - Proportion of pregnant women attending at least four antenatal care visits, routine - Proportion of deliveries taking place in health facilities, routine - Proportion of children with Acute Respiratory Tract Infection (ARI) seeking appropriate health care - Proportion of Newborns who received postnatal care within 48 hours after birth - Proportion of deliveries assisted by skilled attendants’ routine | | |
|  | Early Learning | - Proportion of children 3-5 years participating in formal learning at ECD centers - Proportion of children 3-5 years participating in formal learning at pre-primary school - Proportion of schools that provide school meals - Proportion of villages/mtaa with ECD centers | | |
|  | Responsive caregiving | - Proportion of children 0 - 59 months receiving stimulation for early learning from caregivers - Proportion of children 36-59 months who are developmentally on track - Proportion of health facilities providing comprehensive ECD services for young children | | |
|  | Safety and security | - Proportion of children 0 - 8 years who have their birth registered - Proportion of council hospitals with one-stop centers that provide safety and security services for young children - Proportion of households with access to improved toilets - Proportion of households with access to safe water | | |
| The World Health Organization’s Global Health Observatory (WHO-GHO) database | Health and nutrition | - Childhood immunization coverage index; Immunization spending, Health workers density, Polio immunization coverage, Pentavalent immunization coverage, Measles immunization coverage, Bacillus Calmette–Guerin (BCG) immunization coverage | | |
| The National Evaluation Platform (NEP) | Health and nutrition | - Maternal and child health | | |
| NeoTree application | Health | - Clinical decision support: Emergency decision report and non-emergency decision report | | |
| TIER. Net (Three Integrated Electronic Registers) | Health and nutrition | - Diagnosis and treatment data - NCDs, HIV | | |
| Birth history methods | Health and nutrition | - Immunizations: DPT3 immunization, Measles immunization, Pentavalent immunization. Other key maternal and child health interventions Include Antenatal care, one visit, Exclusive breastfeeding, and Childhood underweight. | | |
| Multiple Indicator Cluster Survey (MICS) | Health | - percentage of children (under age 5) with acute respiratory infection symptoms for whom advice or treatment was sought from a health facility or provider - Percentage of children (under age 5) who slept under an insecticide-treated mosquito net the night prior to the survey - Percentage of households with at least one insecticide-treated mosquito net (ITN) - Percentage of surviving infants who received the third dose of DTP-containing vaccine - Percentage of children who received the second dose of measles-containing vaccine, as per the national schedule   HIV/AIDS:   - Reported number of children (aged 0-14 years) receiving antiretroviral treatment (ART) | | |
|  | Nutrition | - Exclusive breastfeeding (0-5 months) - Continued breastfeeding (20-23 months) - Height-for-age <-2 SD (stunting), Modelled Estimates - Vitamin A Supplementation, two-dose coverage - Iodized salt consumption (>0 ppm) among all tested households | | |
|  | Opportunities for early learning and responsive caregiving | Literacy-numeracy | Can the child read at least four simple, popular words? (Yes/No)  Does the child know the name and recognize the symbol of all numbers from one to ten? (Yes/No)  Can the child identify or name at least ten letters of the alphabet? (Yes/No) | |
|  |  | Learning/cognition | Does the child follow simple directions on how to do something correctly? (Yes/No)  When given something to do, is the child able to do it independently? (Yes/No) | |
|  |  | Social-emotional | Does the child get along well with other children? (Yes/No)  Does the child kick, bite, or hit other children or adults? (Yes/No) (reverse coded)  Does the child get distracted easily? (Yes/No) (reverse coded) | |
|  |  | Physical | Can the child pick up a small object with two fingers, like a stick or a rock, from the ground? (Yes/No)  Is the child sometimes too sick to play? (Yes/No) (reverse coded) | |
|  |  | Others | - Percentage of children aged 36-59 months attending an early childhood education programme - Percentage of children (aged 24-59 months) whose father has engaged in 4 or more activities to provide early stimulation and responsive care in the last 3 days - Percentage of children under age five who have three or more children's books - The percentage of children under age 5 left alone or under the supervision of another child younger than 10 years of age for more than 1 hour at least once in the last week. | |
|  | Safety and security | Wealth index | - Wealth index: (1) car; (2) motorboat; (3) scooter; (4) cart; (5) bicycle; (6) television; (7) refrigerator; (8) radio; (9) watch; (10) mobile telephone; (11) landline telephone; and (12) electrification of the household, | |
|  |  | Child protection | - Percentage of children under age five whose births are registered - Percentage of children (aged 1-14 years) who experienced any physical punishment and/or psychological aggression by caregivers | |
|  |  | Child survival | - Under-five mortality rate (Deaths per 1,000 live births) - Under-five deaths (Number of deaths) - Infant mortality rate (Deaths per 1,000 live births) - Neonatal mortality rate (Deaths per 1,000 live births) - Under-five mortality rate (Female) (Deaths per 1,000 live births) - Under-five mortality rate (Male) (Deaths per 1,000 live births) | |
|  |  | Sanitation | - Proportion of population using safely managed sanitation services - Proportion of population using basic sanitation services - Proportion of population using at least basic sanitation services - Proportion of population using limited sanitation services | |
|  |  | Drinking water | - Proportion of population using safely managed drinking water services - Proportion of population using basic drinking water services - Proportion of population using at least basic drinking water services - Proportion of population using limited drinking water services - Proportion of population using unimproved drinking water sources - Proportion of population using surface water | |
| Health information system | Health and nutrition | - Diagnosis and treatment-related data | | |
| Health data digitalization (HDD) | Health and nutrition | - Diagnosis and treatment-related data | | |
| HearScreenTM application | Health | - Hearing test/screen | | |
| Saving Little Lives (SLL) | Responsive caregiving | - Uptake of Kangaroo Mother Care (KMC) and provision of skin-to-skin contact (responsive caregiving) | | |
| Ghana's Education Management Information System | Opportunities for early learning | - Registration of schools | | |
| Ecdinfohub.org | Opportunities for early learning | - Number of Children aged 0 - 4 in South Africa - Percentage of 0 - 4 Children over S.A. Child Population - Number of Early Learning Programmers (ELPs) - Number of Staff Employed in ELPs - Number of Children enrolled in ELPs - Average number of Children per ELP - Average number of Teaching Staff per ELP - Percentage of Teaching staff with relevant NQF Qualification - Percentage of Teaching Staff with No Qualification - Percentage of ELPs who do not have access to an outdoor playground with suitable equipment - Percentage of ELPS who have age-appropriate books for different age groups - Percentage of ELPs who provide Breakfast - Percentage of ELPs who provide Lunch - ELPs who have Fees as their primary source of funding - The average monthly fee per child | | |
| Suite of Early Learning Measurement Tools (ELOM) | Opportunities for early learning | - Physical stunting, socio-emotional functioning and learning outcomes | | |
| The UNESCO Institute for Statistics (UIS)  The UNESCO Institute for Statistics (UIS) — see SSA section above for full description of design principles and opportunities for a future ECD platform. | Opportunities for early learning | - The percentage of children and young people achieving minimum proficiency levels in reading and mathematics at different stages of education | | |
