# Supplementary material for: Mapping early childhood development data systems in Kenya and sub-Saharan Africa: a scoping review
Source: Front Public Health. 2026 Oct 22;14:1936644. doi: 10.3389/fpubh.2026.1936644 (PMC13736838; doi:10.3389/fpubh.2026.1936644)
Supplement: Supplementary file 3 [file Table_2.docx]

**Supplementary File E**

**Supplementary Table S2: Indicators from ECD data sources and platforms in Kenya**

Supplementary material accompanying: Mapping Early Childhood Development Data Systems in Kenya and Sub-Saharan Africa: A Scoping Review.

| **Data systems/platforms** | **County-specific/National data systems** | **Nurturing Care Framework** | **Indicators** |
| --- | --- | --- | --- |
| Kenya Health Information System (DHIS-2)  Kenya Electronic Medical Records System (EMR)  Antenatal, Intrapartum and Postnatal Care COVID-19 Surveillance Study (ANCOV) | National | Health and Nutrition | Neonatal death, antepartum stillbirth, intrapartum stillbirth, neonatal invasive bloodstream infection, preterm birth, low birth weight, and small-for-gestational-age. |
| Electronic Health Record System (Afya EHMS) | National | Health and Nutrition | Not specified |
| Kenya Health Information System (DHIS-2) | National | Health and Nutrition | - Maternal health care - Vaccination and the health of children - Children's nutrition - Child's size and weight at birth - Nutritional status of children - Breastfeeding status according to age - Infant feeding practices by age - Stunting in children |
| Kenya Electronic Medical Records System (EMR) | National | Health and Nutrition | - % of children under < 5yrs with symptoms of acute respiratory infection who received antibiotics / referred to a health facility; - % of children under < 5yrs with confirmed malaria diagnosis who received treatment; - Underweight among children below five years; - Stunting among children below five years; - Immunization coverage; - Under-five mortality rate (per 1,000 births); - Infant mortality rate (per 1,000 births); - Neonatal mortality rate (per 1,000 births); - % of children under < 5yrs with fever who were tested for malaria; - % of children < 5 years who had diarrhea who were given ORS packets and zinc supplements |
| NEMIS/KEMIS | National | Opportunities for early learning and responsive caregiving | - Name the School Category, Learner sex composition of the school, School Ownership, Legal status, Curriculum offered, Enrolment, Number of age-based learners with disability by grade/form, type of disability and sex, number of staff and their qualifications, facilities, special needs, financial information (revenue and expenditure), attendance, transition, learning materials, WASH facilities, teacher numbers, teacher pupil ratio, teacher. |
| Kenya Demographic Health Survey | National |  | - Early childhood development - Early Childhood Development Index 2030 |
| Civil Registration and Vital Statistics | National | Safety and security | - Birth and death registration |
| Child Protection Management Information System | National |  | - Violence against children, caregiving, adoption, and foster care |
| Kenya National Bureau of Statistics and the National Treasury of Kenya on resource allocation | National | Resource allocation | - Finance, population, education |
| Kenya Open Data Platform | National |  | - Agriculture, health and social data, economy and finance, education, environment, and water and sanitation |
| The Open County Dashboard | National |  | - Agriculture, rural development, health, security, education, energy, science and technology, and budget indicators |
| Nairobi Urban Health and Demographic Surveillance System (NUHDSS) | Nairobi City County [Note: Data for Nairobi and Isiolo counties were available only for the Health and Nutrition NCF domain. No county-level data were found for responsive caregiving, early learning, safety and security, or ECD resourcing. These findings could alternatively be integrated into the Kenya-level NCF domain subsections above.] | Health and Nutrition | Pregnancy and birth registration, Vaccination |
| Standardized Monitoring and Assessment of Relief and Transition (SMART) | Isiolo County | Health and Nutrition | Prevalence of malnutrition among children aged 6 - 59 months and women of reproductive age (15-49 Years), the nutrition status of children 6 to 59 months, the nutritional status of women of reproductive age (15-49 years) based on maternal mid upper arm circumference, immunization coverage; measles (9-59 months), OPV1/3 and Vitamin A supplementation for children aged 6-59 months. |
| Maternal and Child Health (MCH) booklet | National | Health and Nutrition | - Health (caregiver reports on child health status (e.g., illness symptoms, health-seeking behavior)) - Nutrition (feeding practices, breastfeeding, and dietary diversity) - Responsive caregiving (caregiver-child interactions using the Family Care Indicators) - Opportunities for early learning (availability and engagement in early learning activities (e.g., reading, playing, counting)) - Safety and security (household conditions and caregiver supervision) |
| Integrated Community Case Management (iCCM) | National | Health and Nutrition | - % of children under < 5yrs with symptoms of acute respiratory infection who received antibiotics / referred to a health facility; - % of children under < 5yrs with confirmed malaria diagnosis who received treatment; - Underweight among children below five years; - Stunting among children below five years; - Immunization coverage; - Under-five mortality rate (per 1,000 births); - Infant mortality rate (per 1,000 births); - Neonatal mortality rate (per 1,000 births); - % of children under < 5yrs with fever who were tested for - malaria; - % Of children < 5 years who had diarrhea who were given ORS packets and zinc supplements |
| Pediatric Index Testing Cascade in Homa Bay, Kenya | Homa-Bay County | Health | - Children on antiretroviral therapy (ART) |
